# IGF-1R targeting in cancer – does sub-cellular localization matter?

**DOI:** 10.1186/s13046-023-02850-7

**Published:** 2023-10-20

**Authors:** Upendra K. Soni, Liam Jenny, Rashmi S. Hegde

**Affiliations:** Division of Developmental Biology, Department of Pediatrics, Cincinnati Children’s Hospital Medical Center, University of Cincinnati College of Medicine, Cincinnati, OH USA

**Keywords:** IGF-1R, Therapeutics, cancer, Sarcoma, Biomarkers, Combination therapy, Nuclear IGF-1R, Replication stress

## Abstract

The insulin-like growth factor receptor (IGF-1R) was among the most intensively pursued kinase targets in oncology. However, even after a slew of small-molecule and antibody therapeutics reached clinical trials for a range of solid tumors, the initial promise remains unfulfilled. Mechanisms of resistance to, and toxicities resulting from, IGF-1R-targeted drugs are well-catalogued, and there is general appreciation of the fact that a lack of biomarker-based patient stratification was a limitation of previous clinical trials. But no next-generation therapeutic strategies have yet successfully exploited this understanding in the clinic.

Currently there is emerging interest in re-visiting IGF-1R targeted therapeutics in combination-treatment protocols with predictive biomarker-driven patient-stratification. One such biomarker that emerged from early clinical trials is the sub-cellular localization of IGF-1R. After providing some background on IGF-1R, its drugging history, and the trials that led to the termination of drug development for this target, we look more deeply into the correlation between sub-cellular localization of IGF-1R and susceptibility to various classes of IGF-1R - targeted agents.

**Supplementary Information:**

The online version contains supplementary material available at 10.1186/s13046-023-02850-7.

## Background

Kinase signaling pathways drive many of the defining phenotypes of tumor cells, and thus represent attractive targets for therapeutic intervention. The use of kinase inhibitors in oncology is most successful when the kinase target is constitutively activated by gene mutation and patients can be stratified through molecular profiling [1]. The Insulin-like growth factor 1 receptor (IGF-1R), one of the most intensely investigated kinase targets, is neither mutated in cancers nor did the clinical trials use a molecular-profile based stratification of patients. No IGF-1R targeted agent is currently approved for any oncology indication. But our understanding of the IGF-1R signaling cascade, its interplay with other cellular signaling pathways, and non-canonical functions of IGF-1R has now reached the point where a re-examination of IGF-1R as a target for cancer therapeutics could be productive.

**Fig. 1:**
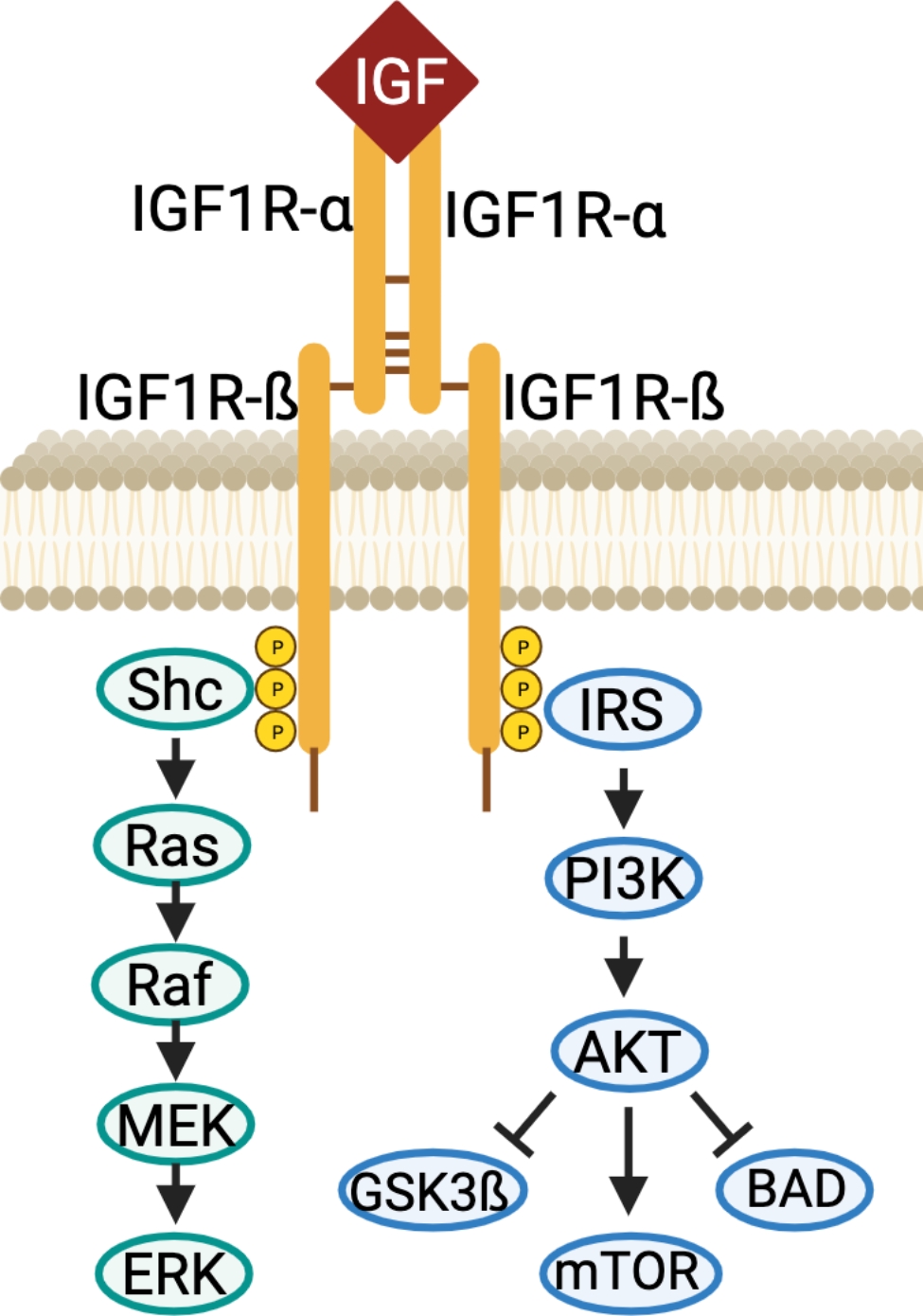
A schematic representation of IGF-1R protein architecture and the signaling cascades activated by the IGF-1R – IGF-1 interaction. Created with BioRender.com

IGF-1R is a receptor tyrosine kinase (RTK) belonging to the insulin receptor family. It is synthesized as a 180 kDa precursor that is then processed to form the mature α_2_ß_2_ receptor (a dimer of two aß subunits held together by disulfide bonds) (Fig. 1). The extra-cellular domain consists of the α-chain and 195-residues of the ß-chain. The rest of the ß-chain contains a single-pass transmembrane domain and a cytoplasmic tyrosine kinase domain. Unlike many other receptor tyrosine kinases, dimerization is not a mechanism of activation for the IGF-1R family. Rather, ligand-binding induces conformational changes of the pre-formed α_2_ß_2_ hetero-tetramer leading to autophosphorylation of the intracellular domain and the creation of docking sites for signaling molecules. This in turn activates the PI3K-AKT-mTOR and the RAS-MAPK signaling cascades, variously promoting cell proliferation, anti-apoptosis, metabolism, differentiation, and cell motility. IGF-1R is ubiquitously expressed and contributes to normal tissue growth [2]. In vivo the relative expression of IGF-1R and the related insulin receptor (InsR) can vary among tissues and stages of development, with commensurate differences in the role of the ligands IGF-1, IGF-2 and insulin on the regulation of metabolic function and growth.

The related InsR has two isoforms: InsR-A and InsR-B. In addition to the α_2_ß_2_ dimers described above, αß monomers of IGF-1R and InsR can form hybrid InsR/IGF-1R heterodimers that are signaling-competent [3, 4]. The ratio of hybrid versus IGF-1R and InsR homodimeric receptors in any given tissue is influenced by the relative concentrations of each receptor, as well as being modulated by other factors such as diet and obesity [5, 6]. A third member of this receptor family, IGF-2R, does not have a kinase domain and does not activate downstream signaling. IGF-2R dampens IGF-1R signaling by sequestering the ligand IGF-2 [7].

While the ligands IGF-1, IGF-2, and insulin have the highest affinity towards their cognate receptors, they can also bind and activate other receptors in the family (for instance IGF-1 can bind IGF-1R and InsR/IGF-1R, IGF-2 can bind IGF-2R, InsR-A, InsR/IGF-1R and IGF-1R, insulin can bind InsR and InsR/IGF-1R) [8]. Despite these overlaps there are differences in downstream effects, with IGF-1R preferentially mediating cell growth and InsR preferentially regulating metabolism. Furthermore, IGF-1R is also able to associate with several other receptors. For instance, IGF-1R – integrin complexes are reported at sites of focal adhesion [9] and IGF-1R – E-cadherin complexes play a role in cell-cell adhesion [10]. Epidermal growth factor EGFR can also interact with IGF-1R, and loss of EGFR leads to depletion of IGF-1R [11]. Even without direct receptor-receptor interaction, crosstalk between IGF-1R and other receptors can occur when receptors in proximity on the membrane influence each other’s signaling. This type of interaction has been reported between IGF-1R and thyrotropin receptor (TSHR) [12]. And finally, crosstalk between IGF-1R and G-protein coupled receptor (GPCR) signaling pathways is mediated by their convergence upon mTORC1 activation [13].

IGF-1R - mediated cellular signaling is thus part of a complex web of well-recognized downstream molecular cascades and crosstalk between members of the IGF-1R family, other RTKs, adhesion receptors and GPCR-induced signaling. Such complexity undoubtedly contributes to both the side-effects of IGF-1R targeted therapeutics and to mechanisms of resistance.

### IGF-1R – in cancer

The IGF1 - IGF-1R signaling pathway regulates numerous cellular phenotypes associated with tumor cell survival and growth – including cell cycle progression, apoptosis and differentiation [14, 15]. Other cancer-linked phenotypes regulated by IGF-1R signaling include cell adhesion and migration [16], cancer metastasis [17, 18], anchorage-independent growth [19], tumor angiogenesis [20], and the epithelial to mesenchymal transition [21]. Elevated levels or activation of IGF-1R also confer resistance to chemotherapeutics [22, 23] and radiation [24].

While the classical InsR-B is primarily involved in control of glucose uptake and metabolism [25], InsR-A (normally expressed in fetal tissues) promotes mitosis, cell invasion and protection from apoptosis upon IGF-2 stimulation in a variety of cancers [26–32]. The IGF-1R/InsR heterodimers in malignant cells are predominantly composed of InsR-A, and can bind IGF-1, IGF-2 and insulin [33].

There is a strong positive association between high levels of IGF-1R pathway signaling and cancer [34], though activating mutations of the *IGF-1R* gene have not been reported. High levels of IGF-1R are seen on the membrane and/or the cytoplasm, and in the nucleus of numerous cancer cell types including prostate cancer [35], head and neck squamous cell cancer [36], breast cancer [37], pancreatic cancer [38], colorectal cancer [39], non-small cell lung cancer [40], Ewing sarcoma [41], and osteosarcoma [42]. High circulating levels of the IGF-1R ligand IGF-1 can also activate IGF-1R signaling and is correlated with increased risk of prostate [43], ovarian [44], and breast cancer [45], as well as possibly second primary cancers [46]. Furthermore stroma-derived IGF-2 is associated with colon cancer progression [47].

### IGF-1R – in cancer therapeutics

Not surprisingly, the IGF-1R signaling pathway has attracted intense interest from the drug development community. According to one recent analysis, between 2003 and 2021 16 IGF-1R inhibitors (Table 1) entered a total of 183 oncology clinical trials involving 12,396 patients [48]. Remarkably none of these drugs obtained approval for use in cancer treatment!

**Table 1:** IGF-1R targeted drugs evaluated in human trials

DRUG	CLASS
AMG479 (Ganitumab)	Human monoclonal antibody targeting IGF-1R
AVE1642	Human monoclonal antibody targeting IGF-1R
BIIB022	Human monoclonal antibody targeting IGF-1R
CP-751,871 (figitumab)	Human monoclonal antibody targeting IGF-1R
IMCA12 (cixutumumab)	Human monoclonal antibody targeting IGF-1R
MK7454 (robatumumab)	Human monoclonal antibody targeting IGF-1R
MK0646 (dalotuzumab)	Human monoclonal antibody targeting IGF-1R
MM141 (istiratumab)	Human monoclonal bispecific antibody targeting IGF-1R and ErbB3
RG1507 (teprotumumab)	Human monoclonal antibody targeting IGF-1R
AXL1717 (picropodophyllotoxin)	Small molecule non ATP-competitive IGF-1R kinase inhibitor
BMS-754,807	Small molecule ATP-competitive IGF-1R kinase inhibitor
KW-2450	Small molecule IGF-1R and InsR kinase inhibitor
OSI906 (Linsitinib)	Small molecule ATP-competitive IGF-1R and InsR kinase inhibitor
PL225B	Small molecule IGF-1R kinase inhibitor
XL228	Multi-targeted small molecule inhibitor of the IGF1R, Src, Abl tyrosine kinases
IGV-001	Biologic-device; patient derived GBM cells treated with an antisense oligodeoxynucleotide towards IGF-1R

Among the first IGF-1R pathway targeted agents were monoclonal antibodies that block receptor-ligand interactions and thus activation of the downstream signaling pathways. These showed promise in pre-clinical studies, and three of them (ganitumab, figitumumab, dalotuzumab) were tested in Phase III trials. Figitumab with chemotherapy did not improve progression-free survival (PFS) in a trial with advanced non-small-cell lung cancer [49]. Dalotuzumab did not yield promising results in a trial with chemo-refractory *KRAS* wild-type metastatic colon cancer [50] (though there was some promise evident in a single Ewing sarcoma patient treated with dalotuzumab [51]). Ganitumab did not improve overall survival (OS) of patients with metastatic adenocarcinoma of the pancreas when tested in combination with Gemcitabine [52]. A Phase III trial of ganitumab combined with interval-compressed chemotherapy recently concluded without evidence of event-free survival (EFS) in patients with metastatic Ewing sarcoma [53].

Two antibodies towards the IGF-1R ligands were tested in humans - dusigitumab and xentuzumab. Xentuzumab, a monoclonal antibody that binds to both IGF-1 and IGF-2, showed initial promise [54]. However, two recently concluded trials in prostate [55] and metastatic breast [56] cancer yielded disappointing results.

Both ATP competitive and ATP non-competitive tyrosine kinase inhibitors (TKI) were developed towards IGF-1R (Table 1) and showed remarkable efficacy in pre-clinical studies. However, because there is a high degree of similarity in the sequence and structure of the kinase domains of IGF-1R and InsR, most ATP-competitive inhibitors inhibited both receptors. Inhibition of the InsR is associated with hyperinsulinemia and hyperglycemia.

The most extensively studied IGF-1R TKI is the ATP-competitive Linsitinib, but disappointing results were reported in numerous trials. This included gastrointestinal stromal tumors [57], adrenocortical cancer [58], non-small cell lung cancer [59], breast cancer [60], and Ewing sarcoma [61]. AXL-1717 is a non-ATP competitive IGF1R kinase inhibitor that has shown some promise and has orphan drug status for treatment of patients with relapsed malignant astrocytomas [62]. No other small molecule inhibitors of IGF-1R remain in cancer clinical trials.

### IGF-1R therapeutics – reasons for their limited success in oncology

Remarkably, after many decades of effort in developing IGF-1R targeted therapeutics for oncological indications (at an estimated cost of $1.63 billion for industry trials [48]), the only FDA-approved drug is the antibody teprotumumab (Tepezza®) for the treatment of thyroid eye disease [63]. Linsitinib is also in a Phase 2b trial for this ophthalmological indication [64]. Although a couple of clinical trials examining combination treatment regimens remain active, no IGF-1R-targeting agent has yet been approved for use in cancer. The failure of IGF-1R-pathway blocking therapeutics in the oncology space has been the subject of much handwringing [65–71], as well as research into the causes underlying clinical failure of agents that showed pre-clinical promise.

Some of the most frequently cited reasons for the failure of IGF-1R-targeted treatments are listed in Table 2. Lack of clinical efficacy is most often attributed to the upregulation of compensatory signaling pathways in response to IGF-1R pathway inhibition. This is not surprising. As with most RTKs, IGF-1R acts as a node in a complex web of “robust” signaling networks [72, 73]. Crosstalk between IGF-1R and other receptors (e.g. EGFR [11], integrins [74–76], GPCR signaling components [77], InsR-A [78]) contribute to the complexity (and failure) that has plagued IGF-1R targeted drug development, but also offer more possibilities for co-targeting strategies. Success in countering such complex signaling systems will likely require multi-component therapy. A listing of some of the combination treatment approaches that have been tested is provided in Table 3.

**Table 2:** Common mechanisms of resistance to IGF-1R -targeted drugs

Mechanisms of resistance to IGF-1R targeted therapy	References
Constitutive activation of downstream signaling PI3K-AKT-mTOR	[85, 140]
Constitutive activation of downstream signaling MAPK cascade	[141]
Absence of IRS-1, IRS-2	[142]
Plasma IGF-1R sequestering of anti-IGF1R antibodies	[143]
InsR signaling compensating for IGF1R inhibition	[144]
InsR-IGF1R hybrid receptors	[28, 145]
Crosstalk with other receptors	[11, 74, 75, 146–149]

**Table 3:** A sampling of the combinations of IGF-1R targeted drugs and other targeted agents that have entered clinical trials

IGF-1R inhibitor	Co-targeted protein	Partner drug	Tumors	NCT Number
Ganitumab	mTOR	Evorolimus	Advanced cancer	NCT01061788
R1507	mTOR	Evorolimus	Advanced solid tumors	NCT00985374
Cixutumumab	mTOR	Everolimus	Neuroendocrine carcinoma	NCT01204476
Cixutumumab	mTOR	Everolimus	Solid tumors, NSCLC	NCT01061788
Cixutumumab	mTOR	Temsirolumus	Pediatric solid tumors	NCT00880282
Cixutumumab	mTOR	Temsirolumus	Sarcoma	NCT01614795
Cixutumumab	mTOR	Temsirolumus	Sarcoma	NCT01016015
Cixutumumab	mTOR	Temsirolumus	Metastatic prostate cancer	NCT01026623
Cixutumumab	mTOR	Temsirolumus	Breast cancer	NCT00699491
AVE1642	proteasome	Bortezomib	Multiple myeloma	NCT01233895
OSI-906	EGFR	Erlotinib	Breast cancer	NCT01205685
OSI-906	EGFR	Erlotinib	Metastatic breast cancer	NCT01013506
AVE1642	EGFR	Erlotinib	Liver carcinoma	NCT00791544
Cixutumumab	EGFR	Erlotinib	NSCLC	NCT00778167
Cixutumumab	EGFR	Erlotinib	Pancreatic cancer	NCT00617708
Ganitumab	HER-2	Trastuzumab	Breast cancer	NCT01479179
Cixutumumab	EGFR/	Lapatinib	Breast cancer	NCT00684983
Cixutumumab	MEK-1/2	Selumetinib	Adult solid neoplasms	NCT01061749
Ganitumab	SFK	Dasatinib	Rhabdomyosarcoma	NCT03041701
Figitumumab	GH	Pegvisomant	Advanced solid tumors	NCT00976508
Ganitumab	CDK4/6	Pablociclib	Relapsed Ewsing sarcoma	NCT04129151
BIIB022	Kinases	Sorafenib	Hepatocellular carcinoma	NCT00956436
AVE164	Kinases	Sorafenib + Erlotinib)	Metastatic liver cancer	NCT00791544

Many RTKs engage the same receptor-proximal signal-transduction pathways as IGF-1R – notably the PI3K-AKT-mTOR and Ras-MAPK pathways. Hence compensatory upregulation of another RTK could counteract IGF-1R inhibition. For instance, in adrenocortical carcinoma IGF-1R inhibition with the kinase inhibitor NVP-AEW541 was found to induce compensatory activation of ERK and sustained mTOR activation, possibly via EGFR [79]. Co-targeting of IGF-1R (Dalotuzumab) and EGFR (Erlotinib[80], Cetuximab [50]) however did not improve outcomes. In another feedback loop, IGF-1R antibodies elevate levels of growth hormone (GH), IGF-1 and insulin [81, 82]. GH activates oncogenic Akt, PI3K and MAPK activity [83] thus countering the effect of IGF-1R inhibition. High IGF-1 levels could compete for binding to the IGF-1R thus reversing the inhibitory effect of IGF-1R-targeted antibodies [84].

Another contributor to resistance towards IGF-1R targeted agents could be constitutive activation of a downstream IGF-1R effector. For instance, AKT is activated due to PI3K mutations or PTEN deletions frequently found in cancer patients [85–87]. Further, AKT and mTOR inhibitors are known to upregulate IGF-1R through feedback loops [88–90]. A phase I trial of combined dalotuzumab with the AKT inhibitor MK-2206 showed tolerability [91], but no efficacy information is available. The IGF-1/IGF-1R/PI3K/AKT/mTOR cascade remains attractive for therapy especially in protocols that co-target two or more proteins in the pathway.

In other combinations, ganitumab was evaluated with the cyclin-dependent kinase (CDK) inhibitor pablociclib in patients with relapsed Ewing sarcoma, but no therapeutic benefit was reported [92]. Evaluation of ganitumab with the Src kinase inhibitor dasatinib in rhabdomyosarcoma [93] had to be terminated when the drug was discontinued.

The hyperglycemia that results from simultaneous inhibition of InsR signaling could be overcome through the design of more selective inhibition strategies, for instance inhibitors that target IGF-1R over InsR (perhaps through allosteric mechanisms). Another approach is inhibition of the IGF-1R signal adapters IRS-1 and IRS-2. Management of hyperglycemia through co-administration of Metformin has shown promise in trials with figitumumab or BMS-754,807 [94, 95].

In addition to these molecular mechanism-based rationale for the limited success of IGF-1R therapeutics, trial design also played a role. In all early-stage clinical studies of IGF-1R therapeutics, there were always a few patients for whom there was substantial and long-lasting benefit, whetting the appetite for additional study. But later trials never lived up to the promise, or further trials were not pursued because pharmaceutical development of the drug was terminated. Most of the clinical trials were also performed on unstratified trial participants, and for the most part trials did not obtain pre-treatment tumor biopsies that could have been useful in biomarker development. In contrast, most successful clinical trials for targeted anticancer agents use predictive biomarkers in patient selection [96].

With the currently renewed interest in evaluating IGF-1R - targeted therapeutics that are on the shelf due to lack of efficacy despite being tolerable and safe, several promising biomarkers have emerged. These include the ligands IGF-1 and IGF-2, the IGF binding proteins IGFBPs that regulate the bioavailability of the IGFs, the level of the IGF-1R receptor and its localization, and the IRS-1 and IRS-2 adaptor proteins [97, 98]. Many of these biomarkers emerged from pre-clinical studies. The few analyses of clinical data that are available paint a complicated (or incomplete) picture. For instance, post-hoc analyses of pre- and post-treatment biopsy samples from Ewing sarcoma patients treated with either IGF-1R mAb or a combination of cixutumumab and the mTOR inhibitor temsirolimus revealed that median PFS and OS was better in phospho-IGF-1R-negative patients. However, total IGF-1R did not predict outcomes [99]. In metastatic pancreatic cancer patients treated with ganitumab and gemcitabine, higher circulating levels of IGF-1, IGF-2 or IGFBP-3 was associated with better response in Phase II trials [100], but stratifying patients based on these biomarkers did not translate into survival benefit in Phase III trials [52]. Additional analyses of the molecular profiles of tumors from patients who are either responsive or non-responsive to specific IGF-1R-targeted therapeutics will greatly benefit future stratified trials.

### IGF-1R – heading to the nucleus

Receptor tyrosine kinase signaling is typically regulated both by ligand-binding and endocytosis. In keeping with this canonical model, IGF-1R can undergo endocytosis upon ligand-binding. Receptor internalization is initiated by vesicle formation on the membrane and endocytosis via either clathrin-coated pits [101] or the formation of lipid rafts (calveolae) [102]. Once in the early endosome, IGF-1R can be targeted for degradation [103], recycled back to the plasma membrane [104], transported to either the Golgi apparatus [105] or to the nucleus [106, 107]. While receptor degradation allows for termination of signaling, recycling back to the plasma membrane is a mechanism for sustained signaling. IGF-1R in the Golgi is commonly seen in migratory cancer cells [105].

The present review is specifically focused on nuclear IGF-1R and how this subcellular localization might impact sensitivity to IGF-1R-targeted therapeutics. In most instances of RTK trafficking to the nucleus, an intracellular domain (ICD) fragment of the receptor is generated through proteolytic processing and the ICD then enters the nucleus [108]. There are however cases (including IGF-1R [107, 109], ErbB-1 [110], ErbB-2 [111], Ron [112], FGFR1 and FGFR2 [113], VEGFR1 and R2 [114, 115]) in which the intact receptors translocate to the nucleus.

IGF-1R does not contain a nuclear localization signal (NLS), hence active mechanisms of nuclear import must contribute to nuclear localization. Multiple mechanisms have been proposed including a sumoylation-dependent process in which binding to the largest subunit of the dynactin complex p150^Glued^ facilitates transport of IGF-1R to the nuclear pore complex. Interaction with the transport receptor importin-ß and the nucleoporin RanBP2 then promotes SUMOylation (RanBP2 has a SUMO E3 ligase domain) and nuclear translocation [116]. Other proposed routes to the nucleus include association with IRS-1 [117] and hetero-dimerization with the InsR [118].

Interestingly, several studies report both IGF-1Rα and IGF-1Rß subunits in the nucleus making it the only instance of a multi-subunit membrane receptor that traffics to the nucleus [107, 119].

### IGF-1R – in the nucleus

The functional relevance of nuclear IGF-1R is underscored by reports that it is linked to increased IGF-induced proliferation, resistance to the EGFR inhibitor geftinib, and enhanced tumorigenicity [120–122]. At the molecular level nuclear IGF-1R can impact transcription [106, 118, 123] and promote DNA Damage Tolerance (DDT) [124].

Direct interaction between nuclear IGF-1R and DNA is evidenced by both electrophoretic mobility shift assays [109] and chromatin immunoprecipitation experiments [106]. This facilitates RNAPol2 recruitment at active enhancers and upregulation of gene expression. Target genes include *JUN* and *FAM21* which in turn promote cancer cell survival and migration [106]. IGF-1R is also known to bind the Wnt-signaling associated transcription factor LEF-1 [123], and upregulate TCF-mediated transcriptional activity of ß-catenin [123, 125]. Further, IGF-1R binds to, and stimulates, its cognate promoter thus contributing to autoregulation [126]. The kinase activity of nuclear IGF-1R is also implicated in the phosphorylation of histone H3, recruitment of the ATP-dependent helicase Brg1 and the expression of *SNAI2* [118], which in turn is involved in cell migration and epithelial-mesenchymal transformation.

**Fig. 2:**
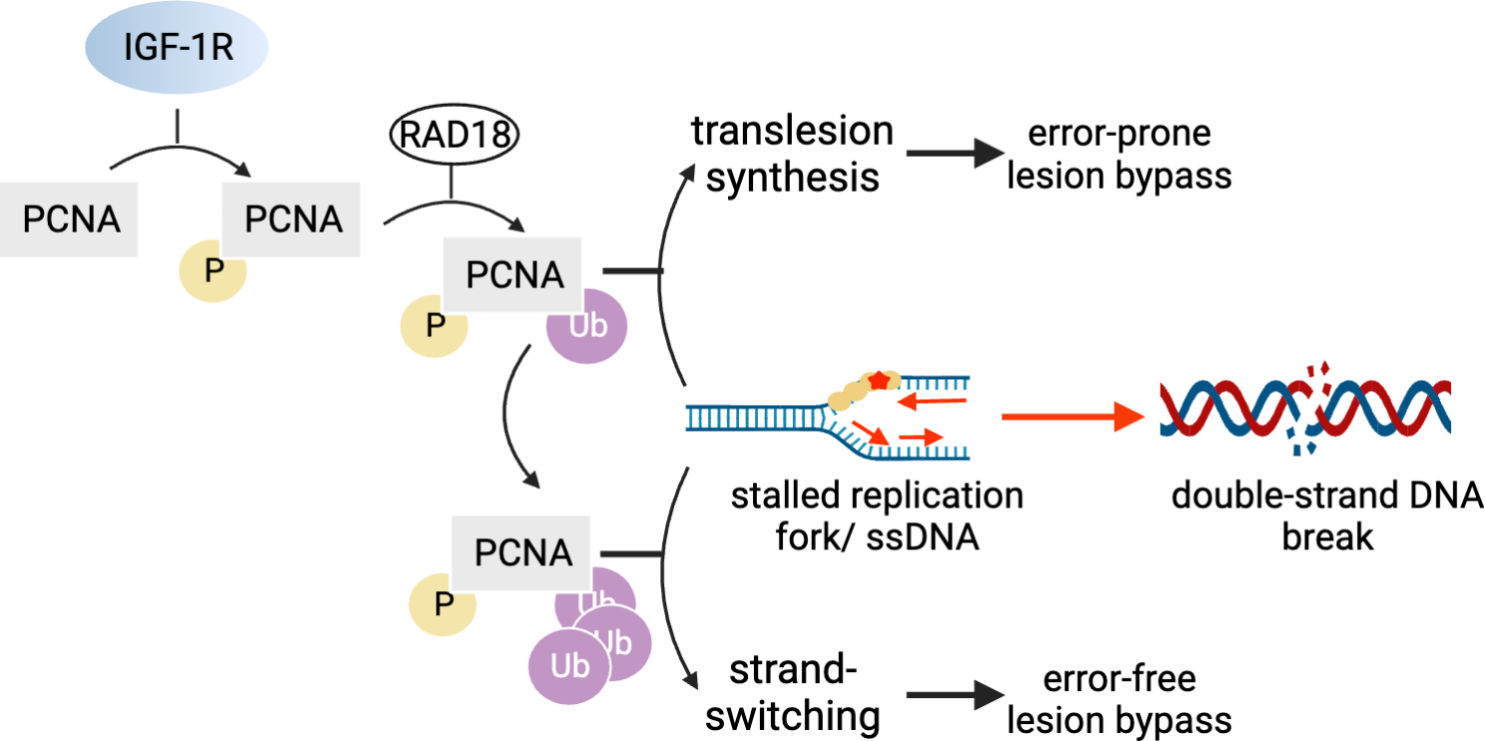
IGF-1R and DNA Damage Tolerance. Nuclear IGF-1R phosphorylates PCNA and promotes ubiquitination by RAD18. Ubiquitinated PCNA promotes recruitment of permissive polymerases to ssDNA breaks and stalled replication forks (markers of replication stress). Mono-ubiquitinated PCNA promotes translesion synthesis and error-prone lesion bypass. Poly-ubiquitinated PCNA promotes strand-switching and an error-free lesion bypass mechanism. In either case, the nuclear IGF-1R - PCNA pathway permits stalled replication to proceed allowing cells survival. Unrepaired ssDNA lesions would lead to double-stranded DNA breaks, and cell death. Hence nuclear IGF-1R promotes the survival of tumor cells under replication stress. Created with BioRender.com.

Independent of its contribution to the regulation of transcription, nuclear IGF-1R also promotes DDT (Fig. 2). DDT is activated when a block in DNA replication (replication stress) uncouples DNA unwinding and synthesis resulting in the formation of single-stranded DNA (ssDNA). Prolonged stalling of replication forks (unrepaired ssDNA breaks) leads to fork collapse and the formation of cytotoxic double-strand DNA breaks. Through DDT, ssDNA breaks are bypassed via either an error-prone mechanism triggered by mono-ubiquitination of proliferating cell nuclear antigen (PCNA) and trans-lesion synthesis, or an error-free lesion bypass mechanism involving template switching to the undamaged strand and requiring polyubiquitination of PCNA (Fig. 2). Hence DDT permits survival of highly proliferative tumor cells in the face of replication stress. Nuclear IGF-1R interacts with, and phosphorylates, PCNA thereby promoting ubiquitination of PCNA by the DDT-dependent E2/E3 ligases [124]. Ubiquitinated PCNA induces the switch to low-fidelity DNA polymerases that allow bypass of DNA lesions thus rescuing stalled replication forks and permitting ongoing DNA replication.

Various components of the DDT pathway are candidate biomarkers of therapy response and clinical outcomes. These include, for example, RAD18 [127, 128], DNA polymerase zeta [129], and DNA polymerase iota [130].

Replication stress in cancer cells can result from endogenous sources (transcription-replication conflicts, nucleotide pool imbalances, ssDNA gas, abasic sites, changes in origin firing frequency), or exogenous triggers such as radiation or chemotherapy. Oncogenes also commonly induce replication stress [131]. Given the positive association between nuclear IGF-1R and DDT, it is not surprising that higher levels of nuclear IGF-1R have recently been correlated with lower levels of endogenous replication stress in cancer cells [132]. Nuclear IGF-1R is a promising biomarker that could identify cells with higher levels of DDT and lower levels of replication stress [132].

### IGF-1R – does nuclear localization affect sensitivity to IGF-1R - targeted drugs?

Sub-cellular localization of IGF-1R (membranous, cytoplasmic, or nuclear) is an attractive candidate prognostic biomarker since it is readily assessable using biopsy tissue. The lack of IGF-1R on the membrane is a reasonable predictor of poor response to IGF-1R mAbs, but small molecule TKIs should still be effective. In general, the presence of nuclear IGF-1R has been linked with worse clinical outcomes. Among colorectal cancer patients nuclear IGF-1R levels were reported to be higher in metastatic tumors relative to paired untreated primary tumors and correlated with a worse prognosis [23]. Curiously, this study also showed that treatment with ganitumab increased the nuclear localization of IGF-1R. A similar association between nuclear IGF-1R and worse outcomes has been reported in osteosarcomas [133], pediatric gliomas [134], synovial sarcomas [135], breast cancer [126], clear cell renal cell carcinoma [107], and embryonal rhabdomyosarcoma [136]. However, in Ewing sarcoma, the sub-cellular localization does not seem to correlate with tumor stage (primary, metastatic, relapsed) [132]. Inhibition of the IGF-1R kinase activity suppresses nuclear translocation of IGF-1R consistent with the model that nuclear translocation is dependent on IGF-1 stimulation [107]. In Ewing sarcoma cells with constitutive nuclear IGF-1R, treatment with the kinase inhibitor Linsitinib reduced the level of ubiquitinated-PCNA, thus likely attenuating DDT [132]. Inhibition of nuclear IGF-1R kinase activity with NVP AEW-541 reduced expression of the transcription target gene *SNAI2* that is associated with cancer cell invasiveness and metastasis [118].

One study associated exclusively nuclear IGF-1R with better progression-free and overall survival in patients with soft tissue sarcomas, Ewing sarcomas and osteosarcomas treated with IGF-1R-directed antibodies [137]. This is curious because of the general assumption that nuclear IGF-1R cannot be accessed by antibodies - crossing the membrane is challenging and import to the nucleus of an antibody bound to IGF-1R is unlikely because of the size of the complex.

Given the preponderance of data that links nuclear IGF-1R to worse outcomes after IGF-1R - directed treatments, inhibiting nuclear translocation of IGF-1R or inhibiting its sequestration in the nucleus could re-sensitize tumor cells to treatment. Reducing levels of nuclear IGF-1R has been achieved by treatment with the IGF-1R kinase inhibitor AZ12253801 [107] or the clathrin inhibitor dansylcadaverine [107]. Intervention strategies such as these have not yet been translated into the clinic.

One notable recent advance is the correlation between nuclear IGF-1R and lower levels of endogenous replication stress in Ewing sarcoma tumor cells [132]. There is evidence that tumors with high endogenous replication stress levels are more sensitive to further replication stress exacerbation by drugs such as gemcitabine, ATR inhibitors or checkpoint inhibitors [138, 139]. The low replication stress/nuclear IGF-1R subset of tumors could be sensitized to gemcitabine by inhibiting nuclear IGF-1R localization, sequestration or activity. Indeed, in pre-clinical studies inhibition of IGF-1R with Linsitinib combined with WEE1 (checkpoint) inhibition led to tumor regression of low replication stress Ewing sarcoma tumors [132]. Such IGF-1R sub-cellular localization-informed strategies are a promising way to stratify patients for treatment with combinations of rational targeted agents.

## Conclusions

The early excitement about IGF-1R - targeted treatments has not translated into the clinical success that one might have anticipated. Certainly, undesirable side-effects arising from crosstalk with the InsR signaling pathway were a concern. Nevertheless, if the anti-tumor activity had been significant the issues with maintaining glucose homeostasis were likely manageable. The clinical studies testing various IGF-1R therapeutics did not stratify patients with any molecular markers. Further, by the time candidate predictive biomarkers were proposed and combinatorial treatment protocols designed to counter compensatory signaling pathways, pharmaceutical development of most IGF-1R targeting agents for oncology had ceased. Evidence summarized in this review support a re-examination of the use of IGF-1R sub-cellular localization as a biomarker in the selection of combination treatment regimens. Combining IGF-1R localization with other protein biomarkers, notably markers of replication stress [132], could enrich the patient population selected for IGF-1R targeted therapeutics. Additionally, this approach can help identify optimum combination drug regimens. A limitation of this approach is one shared by most immunohistochemistry (IHC) methods of biopsy analysis – IHC requires interpretation by expert pathologists and CLIA-certified protocols.

The intense interest in targeting IGF-1R has undoubtedly resulted in a large inventory of potential drugs that either never entered trials or were not pursued further when the disappointing data from unstratified trials emerged. A biomarker-informed re-evaluation of this inventory of drug candidates would be productive.

### Electronic supplementary material

Below is the link to the electronic supplementary material.

Supplementary Material 1

Supplementary Material 2

## Data Availability

Not applicable.

## Abbreviations

IGF-1R: Insulin-like growth factor receptor 1
IGF-2R: Insulin-like growth factor receptor 2
InsR: Insulin receptor
RTK: receptor tyrosine kinase
PI3K: phosphoinositide 3-kinase
AKT: also known as protein kinase B PKB
mTOR: mammalian target of rapamycin
RAS: a family of GTP-binding proteins, the name derives from “Rat sarcoma”
MAPK: mitogen-activated protein kinases
IGF-1, IGF-1: insulin-like growth factor 1 and 2
TSHR: thyrotropin receptor
GPCR: G-protein coupled receptor
PFS: progression-free survival
OS: overall survival
EFS: event-free survival
ATP: adenosine tri-phosphate
TKI: tyrosine kinase inhibitors
EGFR: epidermal growth factor receptor. Family includes other ErbB receptors
ERK: extracellular signal-related kinase
SFK: Src family kinase
CDK: cyclin-dependent kinase
IRS-1 and IRS-2: insulin receptor substrate 1 and 2
IGFBP: Insulin-like growth factor binding protein
mAb: monoclonal antibody
ICD: intracellular domain
FGFR1 and FGFR2: Fibroblast growth factor receptor 1 and 2
VEGFR1 and R2: vascular endothelial growth factor receptor-1 and − 2
NLS: nuclear localization signal
DDT: DNA Damage Tolerance
RNAPol2: RNA polymerase 2
LEF-1: lymphoid enhancer binding factor1
TCF: T cell factor
ssDNA: single-stranded DNA
PCNA: proliferating cell nuclear antigen
RAD18: A ubiquitin ligase that ubiquitinates PCNA
DNA: deoxyribonucleic acid
GH: Growth hormone
NCT: National clinical trial
IHC: immunohistochemistry
CLIA: Clinical laboratory improvement amendments
