# Supplementary material for: IGF-1R targeting in cancer – does sub-cellular localization matter?
Source: J Exp Clin Cancer Res. 2023 Oct 20;42:273. doi: 10.1186/s13046-023-02850-7 (PMC10588251; doi:10.1186/s13046-023-02850-7)
Supplement: Supplementary file 2 — Supplementary Material 2 [file 13046_2023_2850_MOESM2_ESM.docx]

**IGF-1R targeting in cancer – does sub-cellular localization matter?**

**Upendra K. Soni^1^, Liam Jenny^1^, Rashmi S. Hegde^1^***

**Affiliation:**

^1^Division of Developmental Biology, Department of Pediatrics, Cincinnati Children’s Hospital Medical Center, University of Cincinnati College of Medicine, Cincinnati, OH, USA

*Corresponding author name and email address: Rashmi Hegde - [rashmi.hegde@cchmc.org](mailto:rashmi.hegde@cchmc.org)

**Abstract**

The insulin-like growth factor receptor (IGF-1R) was among the most intensively pursued kinase targets in oncology. However, even after a slew of small-molecule and antibody therapeutics reached clinical trials for a range of solid tumors, the initial promise remains unfulfilled. Mechanisms of resistance to, and toxicities resulting from, IGF-1R-targeted drugs are well-catalogued, and there is general appreciation of the fact that a lack of biomarker-based patient stratification was a limitation of previous clinical trials. But no next-generation therapeutic strategies have yet successfully exploited this understanding in the clinic.

Currently there is emerging interest in re-visiting IGF-1R targeted therapeutics in combination-treatment protocols with predictive biomarker-driven patient-stratification. One such biomarker that emerged from early clinical trials is the sub-cellular localization of IGF-1R. After providing some background on IGF-1R, its drugging history, and the trials that led to the termination of drug development for this target, we look more deeply into the correlation between sub-cellular localization of IGF-1R and susceptibility to various classes of IGF-1R - targeted agents.

**Keywords**

IGF-1R, therapeutics, cancer, sarcoma, biomarkers, combination therapy, nuclear IGF-1R, replication stress

**Background**

Kinase signaling pathways drive many of the defining phenotypes of tumor cells, and thus represent attractive targets for therapeutic intervention. The use of kinase inhibitors in oncology is most successful when the kinase target is constitutively activated by gene mutation and patients can be stratified through molecular profiling (1). The Insulin-like growth factor 1 receptor (IGF-1R), one of the most intensely investigated kinase targets, is neither mutated in cancers nor did the clinical trials use a molecular-profile based stratification of patients. No IGF-1R targeted agent is currently approved for any oncology indication. But our understanding of the IGF-1R signaling cascade, its interplay with other cellular signaling pathways, and non-canonical functions of IGF-1R has now reached the point where a re-examination of IGF-1R as a target for cancer therapeutics could be productive.

IGF-1R is a receptor tyrosine kinase (RTK) belonging to the insulin receptor family. It is synthesized as a 180kDa precursor that is then processed to form the mature α_2_ß_2_ receptor (a dimer of two αß subunits held together by disulfide bonds) (Fig. 1). The extra-cellular domain consists of the α-chain and 195-residues of the ß-chain. The rest of the ß-chain contains a single-pass transmembrane domain and a cytoplasmic tyrosine kinase domain. Unlike many other receptor tyrosine kinases, dimerization is not a mechanism of activation for the IGF-1R family. Rather, ligand-binding induces conformational changes of the pre-formed α_2_ß_2_ hetero-tetramer leading to autophosphorylation of the intracellular domain and the creation of docking sites for signaling molecules. This in turn activates the PI3K-AKT-mTOR and the RAS-MAPK signaling cascades, variously promoting cell proliferation, anti-apoptosis, metabolism, differentiation, and cell motility. IGF-1R is ubiquitously expressed and contributes to normal tissue growth (2). In vivo the relative expression of IGF-1R and the related insulin receptor (InsR) can vary among tissues and stages of development, with commensurate differences in the role of the ligands IGF-1, IGF-2 and insulin on the regulation of metabolic function and growth.

The InsR has two isoforms: InsR-A and InsR-B. In addition to the α_2_ß_2_ dimers described above, aß monomers of IGF-1R and InsR can form hybrid InsR/IGF-1R heterodimers that are signaling-competent (3, 4). The ratio of hybrid versus IGF-1R and InsR homodimeric receptors in any given tissue is influenced by the relative concentrations of each receptor, as well as being modulated by other factors such as diet and obesity (5, 6). A third member of this receptor family, IGF-2R, does not have a kinase domain and does not activate downstream signaling. IGF-2R dampens IGF-1R signaling by sequestering the ligand IGF-2 (7).

While the ligands IGF-1, IGF-2, and insulin have the highest affinity towards their cognate receptors, they can also bind and activate other receptors in the family (for instance IGF-1 can bind IGF-1R and InsR/IGF-1R, IGF-2 can bind IGF-2R, InsR-A, InsR/IGF-1R and IGF-1R, insulin can bind InsR and InsR/IGF-1R) (8). Despite these overlaps there are differences in downstream effects, with IGF-1R preferentially mediating cell growth and InsR preferentially regulating metabolism. Furthermore, IGF-1R is also able to associate with several other receptors. For instance, IGF-1R – integrin complexes are reported at sites of focal adhesion (9) and IGF-1R – E-cadherin complexes play a role in cell-cell adhesion (10). Epidermal growth factor EGFR can also interact with IGF-1R, and loss of EGFR leads to depletion of IGF-1R (11). Even without direct receptor-receptor interaction, crosstalk between IGF-1R and other receptors can occur when receptors in proximity on the membrane influence each other’s signaling. This type of interaction has been reported between IGF-1R and thyrotropin receptor (TSHR) (12). And finally, crosstalk between IGF-1R and G-protein coupled receptor (GPCR) signaling pathways is mediated by their convergence upon mTORC1 activation (13).

IGF-1R - mediated cellular signaling is thus part of a complex web of well-recognized downstream molecular cascades and crosstalk between members of the IGF-1R family, other RTKs, adhesion receptors and GPCR-induced signaling. Such complexity undoubtedly contributes to both the side-effects of IGF-1R targeted therapeutics and to mechanisms of resistance.

**IGF-1R – in cancer**

The IGF1 - IGF-1R signaling pathway regulates numerous cellular phenotypes associated with tumor cell survival and growth – including cell cycle progression, apoptosis and differentiation (14, 15). Other cancer-linked phenotypes regulated by IGF-1R signaling include cell adhesion and migration (16), cancer metastasis (17, 18), anchorage-independent growth (19), tumor angiogenesis (20), and the epithelial to mesenchymal transition (21). Elevated levels or activation of IGF-1R also confer resistance to chemotherapeutics (22, 23) and radiation (24).

While the classical InsR-B is primarily involved in control of glucose uptake and metabolism (25), InsR-A (normally expressed in fetal tissues) promotes mitosis, cell invasion and protection from apoptosis upon IGF-2 stimulation in a variety of cancers (26-32). The IGF-1R/InsR heterodimers in malignant cells are predominantly composed of InsR-A, and can bind IGF-1, IGF-2 and insulin (33).

There is a strong positive association between high levels of IGF-1R pathway signaling and cancer (34), though activating mutations of the *IGF-1R* gene have not been reported. High levels of IGF-1R are seen on the membrane and/or the cytoplasm, and in the nucleus of numerous cancer cell types including prostate cancer (35), head and neck squamous cell cancer (36), breast cancer (37), pancreatic cancer (38), colorectal cancer (39), non-small cell lung cancer (40), Ewing sarcoma (41), and osteosarcoma (42). High circulating levels of the IGF-1R ligand IGF-1 can also activate IGF-1R signaling and is correlated with increased risk of prostate (43) , ovarian (44), and breast cancer (45), as well as possibly second primary cancers (46). Furthermore stroma-derived IGF-2 is associated with colon cancer progression (47).

**IGF-1R – in cancer therapeutics**

Not surprisingly the IGF-1R signaling pathway has attracted intense interest from the drug development community. According to one recent analysis, between 2003 and 2021 16 IGF-1R inhibitors (Table 1) entered a total of 183 oncology clinical trials involving 12,396 patients (48). Remarkably none of these drugs obtained approval for use in cancer treatment!

Among the first IGF-1R pathway targeted agents were monoclonal antibodies that block receptor-ligand interactions and thus activation of the downstream signaling pathways. These showed promise in pre-clinical studies, and three of them (ganitumab, figitumumab, dalotuzumab) were tested in Phase III trials. Figitumab with chemotherapy did not improve progression-free survival (PFS) in a trial with advanced non-small-cell lung cancer (49). Dalotuzumab did not yield promising results in a trial with chemo-refractory *KRAS* wild-type metastatic colon cancer (50) (though there was some promise evident in a single Ewing sarcoma patient treated with dalotuzumab (51)). Ganitumab did not improve overall survival (OS) of patients with metastatic adenocarcinoma of the pancreas when tested in combination with Gemcitabine (52). A Phase III trial of ganitumab combined with interval-compressed chemotherapy recently concluded without evidence of event-free survival (EFS) in patients with metastatic Ewing sarcoma (53).

Two antibodies towards the IGF-1R ligands were tested in humans - dusigitumab and xentuzumab. Xentuzumab, a monoclonal antibody that binds to both IGF-1 and IGF-2, showed initial promise (54). However, two recently concluded trials in prostate (55) and metastatic breast (56) cancer yielded disappointing results.

Both ATP competitive and ATP non-competitive tyrosine kinase inhibitors (TKI) were developed towards IGF-1R (Table 1) and showed remarkable efficacy in pre-clinical studies. However, because there is a high degree of similarity in the sequence and structure of the kinase domains of IGF-1R and InsR, most ATP-competitive inhibitors inhibited both receptors. Inhibition of the InsR is associated with hyperinsulinemia and hyperglycemia.

The most extensively studied IGF-1R TKI is the ATP-competitive Linsitinib, but disappointing results were reported in numerous trials. This included gastrointestinal stromal tumors (57), adrenocortical cancer (58), non-small cell lung cancer (59), breast cancer (60), and Ewing sarcoma (61). AXL-1717 is a non-ATP competitive IGF1R kinase inhibitor that has shown some promise and has orphan drug status for treatment of patients with relapsed malignant astrocytomas (62). No other small molecule inhibitors of IGF-1R remain in cancer clinical trials.

**IGF-1R therapeutics – reasons for their limited success in oncology**

Remarkably, after many decades of effort in developing IGF-1R targeted therapeutics for oncological indications (at an estimated cost of $1.63 billion for industry trials (48)), the only FDA-approved drug is the antibody teprotumumab (Tepezza®) for the treatment of thyroid eye disease (63). Linsitinib is also in a Phase 2b trial for this ophthalmological indication (64). Although a couple of clinical trials examining combination treatment regimens remain active, no IGF-1R-targeting agent has yet been approved for use in cancer. The failure of IGF-1R-pathway blocking therapeutics in the oncology space has been the subject of much handwringing (65-71), as well as research into the causes underlying the clinical failure of agents that showed pre-clinical promise.

Some of the most frequently cited reasons for the failure of IGF-1R-targeted treatments are listed in Table 2. Lack of clinical efficacy is most often attributed to the upregulation of compensatory signaling pathways in response to IGF-1R pathway inhibition. This is not surprising. As with most RTKs, IGF-1R acts as a node in a complex web of “robust” signaling networks (72) (73). Crosstalk between IGF-1R and other receptors (e.g. EGFR (11), integrins (74-76), GPCR signaling components (77), InsR-A (78)) contribute to the complexity (and failure) that has plagued IGF-1R targeted drug development, but also offer more possibilities for co-targeting strategies. Success in countering such complex signaling systems will likely require multi-component therapy. A listing of some of the combination treatment approaches that have been tested is provided in Table 3.

Many RTKs engage the same receptor-proximal signal-transduction pathways as IGF-1R – notably the PI3K-AKT-mTOR and Ras-MAPK pathways. Hence compensatory upregulation of another RTK could counteract IGF-1R inhibition. For instance, in adrenocortical carcinoma IGF-1R inhibition with the kinase inhibitor NVP-AEW541 was found to induce compensatory activation of ERK and sustained mTOR activation, possibly via EGFR (79). Co-targeting of IGF-1R (Dalotuzumab) and EGFR (Erlotinib(80), Cetuximab (50)) however did not improve outcomes. In another feedback loop, IGF-1R antibodies elevate levels of growth hormone (GH), IGF-1 and insulin (81, 82). GH activates oncogenic Akt, PI3K and MAPK activity (83) thus countering the effect of IGF-1R inhibition. High IGF-1 levels could compete for binding to the IGF-1R thus reversing the inhibitory effect of IGF-1R-targeted antibodies (84).

Another contributor to resistance towards IGF-1R targeted agents could be constitutive activation of a downstream IGF-1R effector. For instance, AKT is activated due to PI3K mutations or PTEN deletions frequently found in cancer patients (85-87). Further, AKT and mTOR inhibitors are known to upregulate IGF-1R through feedback loops (88-90). A phase I trial of combined dalotuzumab with the AKT inhibitor MK-2206 showed tolerability (91) but no efficacy information is available. The IGF-1/IGF-1R/PI3K/AKT/mTOR cascade remains attractive for therapy especially in protocols that co-target two or more proteins in the pathway.

In other combinations ganitumab was evaluated in combination with the cyclin-dependent kinase (CDK) inhibitor pablociclib in patients with relapsed Ewing sarcoma, but no therapeutic benefit was reported (92). Evaluation of ganitumab with the Src kinase inhibitor dasatinib in rhabdomyosarcoma (93) had to be terminated when the drug was discontinued.

The hyperglycemia that results from simultaneous inhibition of InsR signaling could be overcome through the design of more selective inhibition strategies, for instance inhibitors that target IGF-1R over InsR (perhaps through allosteric mechanisms). Another approach is inhibition of the IGF-1R signal adapters IRS-1 and IRS-2. Management of hyperglycemia through co-administration of Metformin has shown promise in trials with figitumumab or BMS-754807 (94, 95).

In addition to these molecular mechanism-based rationale for the limited success of IGF-1R therapeutics, trial design also played a role. In all early-stage clinical studies of IGF-1R therapeutics, there were always a few patients for whom there was substantial and long-lasting benefit, whetting the appetite for additional study. But later trials never lived up to the promise, or further trials were not pursued because pharmaceutical development of the drug was terminated. Most of the clinical trials were also performed on unstratified trial participants, and for the most part trials did not obtain pre-treatment tumor biopsies that could have been useful in biomarker development. In contrast, most successful clinical trials for targeted anticancer agents use predictive biomarkers in patient selection (96).

With the currently renewed interest in evaluating IGF-1R - targeted therapeutics that are on the shelf due to lack of efficacy despite being tolerable and safe, several promising biomarkers have emerged. These include the ligands IGF-1 and IGF-2, the IGF binding proteins IGFBPs that regulate the bioavailability of the IGFs, the level of the IGF-1R receptor and its localization, and the IRS-1 and IRS-2 adaptor proteins (97, 98). Many of these biomarkers emerged from pre-clinical studies. The few analyses of clinical data that are available paint a complicated (or incomplete) picture. For instance, post-hoc analyses of pre- and post-treatment biopsy samples from Ewing sarcoma patients treated with either IGF-1R mAb or a combination of cixutumumab and the mTOR inhibitor temsirolimus revealed that median PFS and OS was better in phospho-IGF-1R-negative patients. However, total IGF-1R did not predict outcomes (99). In metastatic pancreatic cancer patients treated with ganitumab and gemcitabine, higher circulating levels of IGF-1, IGF-2 or IGFBP-3 was associated with better response in Phase II trials (100), but stratifying patients based on these biomarkers did not translate into survival benefit in Phase III trials (52). Additional analyses of the molecular profiles of tumors from patients who are either responsive or non-responsive to specific IGF-1R-targeted therapeutics will greatly benefit future stratified trials.

**IGF-1R – heading to the nucleus**

Receptor tyrosine kinase signaling is typically regulated both by ligand-binding and endocytosis. In keeping with this canonical model, IGF-1R can undergo endocytosis upon ligand-binding. Receptor internalization is initiated by vesicle formation on the membrane and endocytosis via either clathrin-coated pits (101) or the formation of lipid rafts (calveolae) (102). Once in the early endosome, IGF-1R can be targeted for degradation (103), recycled back to the plasma membrane (104), transported to either the Golgi apparatus (105) or to the nucleus (106, 107). While receptor degradation allows for termination of signaling, recycling back to the plasma membrane is a mechanism for sustained signaling. IGF-1R in the Golgi is commonly seen in migratory cancer cells (105).

The present review is specifically focused on nuclear IGF-1R and how this subcellular localization might impact sensitivity to IGF-1R-targeted therapeutics. In most instances of RTK trafficking to the nucleus, an intracellular domain (ICD) fragment of the receptor is generated through proteolytic processing and the ICD then enters the nucleus (108). There are however cases (including IGF-1R (107, 109), ErbB-1 (110), ErbB-2 (111), Ron (112), FGFR1 and FGFR2 (113), VEGFR1 and R2 (114, 115)) in which the intact receptors translocate to the nucleus.

IGF-1R does not contain a nuclear localization signal (NLS), hence active mechanisms of nuclear import must contribute to nuclear localization. Multiple mechanisms have been proposed including a sumoylation-dependent process in which binding to the largest subunit of the dynactin complex p150^Glued^ facilitates transport of IGF-1R to the nuclear pore complex. Interaction with the transport receptor importin-ß and the nucleoporin RanBP2 then promotes SUMOylation (RanBP2 has a SUMO E3 ligase domain) and nuclear translocation (116). Other proposed routes to the nucleus include association with IRS-1 (117) and hetero-dimerization with the InsR (118).

Interestingly, several studies report both IGF-1Rα and IGF-1Rß subunits in the nucleus making it the only instance of a multi-subunit membrane receptor that traffics to the nucleus (107, 119).

**IGF-1R – in the nucleus**

The functional relevance of nuclear IGF-1R is underscored by reports that it is linked to increased IGF-induced proliferation, resistance to the EGFR inhibitor geftinib, and enhanced tumorigenicity (120-122). At the molecular level nuclear IGF-1R can impact transcription (106) (118, 123) and promote DNA Damage Tolerance (DDT) (124).

Direct interaction between nuclear IGF-1R and DNA is evidenced by both electrophoretic mobility shift assays (109) and chromatin immunoprecipitation experiments (106). This facilitates RNAPol2 recruitment at active enhancers and upregulation of gene expression. Target genes include *JUN* and *FAM21* which in turn promote cancer cell survival and migration (106). IGF-1R is also known to bind the Wnt-signaling associated transcription factor LEF-1 (123), and upregulate TCF-mediated transcriptional activity of ß-catenin (123, 125). Further, IGF-1R binds to, and stimulates, its cognate promoter thus contributing to autoregulation (126). The kinase activity of nuclear IGF-1R is also implicated in the phosphorylation of histone H3, recruitment of the ATP-dependent helicase Brg1 and the expression of *SNAI2 (118)*, which in turn is involved in cell migration and epithelial-mesenchymal transformation.

Independent of its contribution to the regulation of transcription, nuclear IGF-1R also promotes DDT (Fig. 2). DDT is activated when a block in DNA replication (replication stress) uncouples DNA unwinding and synthesis resulting in the formation of single-stranded DNA (ssDNA). Prolonged stalling of replication forks (unrepaired ssDNA breaks) leads to fork collapse and the formation of cytotoxic double-strand DNA breaks. Through DDT, ssDNA breaks are bypassed via either an error-prone mechanism triggered by mono-ubiquitination of proliferating cell nuclear antigen (PCNA) and trans-lesion synthesis, or an error-free lesion bypass mechanism involving template switching to the undamaged strand and requiring polyubiquitination of PCNA (Fig. 2). Hence DDT permits survival of highly proliferative tumor cells in the face of replication stress. Nuclear IGF-1R interacts with, and phosphorylates, PCNA thereby promoting ubiquitination of PCNA by the DDT-dependent E2/E3 ligases (124). Ubiquitinated PCNA induces the switch to low-fidelity DNA polymerases that allow bypass of DNA lesions thus rescuing stalled replication forks and permitting ongoing DNA replication.

Various components of the DDT pathway are candidate biomarkers of therapy response and clinical outcomes. These include, for example, RAD18 (127, 128), DNA polymerase zeta (129), and DNA polymerase iota (130).

Replication stress in cancer cells can result from endogenous sources (transcription-replication conflicts, nucleotide pool imbalances, ssDNA gas, abasic sites, changes in origin firing frequency), or exogenous triggers such as radiation or chemotherapy. Oncogenes also commonly induce replication stress (131). Given the positive association between nuclear IGF-1R and DDT, it is not surprising that higher levels of nuclear IGF-1R have recently been correlated with lower levels of endogenous replication stress in cancer cells (132). Nuclear IGF-1R is a promising biomarker that could identify cells with higher levels of DDT and lower levels of replication stress (132).

**IGF-1R – does nuclear localization affect sensitivity to IGF-1R - targeted drugs?**

Sub-cellular localization of IGF-1R (membranous, cytoplasmic, or nuclear) is an attractive candidate prognostic biomarker since it is readily assessable using biopsy tissue. The lack of IGF-1R on the membrane is a reasonable predictor of poor response to IGF-1R mAbs, but small molecule TKIs should still be effective. In general, the presence of nuclear IGF-1R has been linked with worse clinical outcomes. Among colorectal cancer patients nuclear IGF-1R levels were reported to be higher in metastatic tumors relative to paired untreated primary tumors and correlated with a worse prognosis (23). Curiously, this study also showed that treatment with ganitumab increased the nuclear localization of IGF-1R. A similar association between nuclear IGF-1R and worse outcomes has been reported in osteosarcomas (133), pediatric gliomas (134), synovial sarcomas (135), breast cancer (126), clear cell renal cell carcinoma (107), and embryonal rhabdomyosarcoma (136). However, in Ewing sarcoma, the sub-cellular localization does not seem to correlate with tumor stage (primary, metastatic, relapsed) (132). Inhibition of the IGF-1R kinase activity suppresses nuclear translocation of IGF-1R consistent with the model that nuclear translocation is dependent on IGF-1 stimulation (107). In Ewing sarcoma cells with constitutive nuclear IGF-1R, treatment with the kinase inhibitor Linsitinib reduced the level of ubiquitinated-PCNA, thus likely attenuating DDT (132). Inhibition of nuclear IGF-1R kinase activity with NVP AEW-541 reduced expression of the transcription target gene *SNAI2* that is associated with cancer cell invasiveness and metastasis (118).

One study associated exclusively nuclear IGF-1R with better progression-free and overall survival in patients with soft tissue sarcomas, Ewing sarcomas and osteosarcomas treated with IGF-1R-directed antibodies (137). This is curious because of the general assumption that nuclear IGF-1R cannot be accessed by antibodies - crossing the membrane is challenging and import to the nucleus of an antibody bound to IGF-1R is unlikely because of the size of the complex.

Given the preponderance of data that links nuclear IGF-1R to worse outcomes after IGF-1R - directed treatments, inhibiting nuclear translocation of IGF-1R or inhibiting its sequestration in the nucleus could re-sensitize tumor cells to treatment. Reducing levels of nuclear IGF-1R has been achieved by treatment with the IGF-1R kinase inhibitor AZ12253801 (107) or the clathrin inhibitor dansylcadaverine (107). Intervention strategies such as these have not yet been translated into the clinic.

One notable recent advance is the correlation between nuclear IGF-1R and lower levels of endogenous replication stress in Ewing sarcoma tumor cells (132). There is evidence that tumors with high endogenous replication stress levels are more sensitive to further replication stress exacerbation by drugs such as gemcitabine, ATR inhibitors or checkpoint inhibitors (138, 139). The low replication stress/nuclear IGF-1R subset of tumors could be sensitized to gemcitabine by inhibiting nuclear IGF-1R localization, sequestration or activity. Indeed, in pre-clinical studies inhibition of IGF-1R with Linsitinib combined with WEE1 (checkpoint) inhibition led to tumor regression of low replication stress Ewing sarcoma tumors (132). Such IGF-1R sub-cellular localization-informed strategies are a promising way to stratify patients for treatment with combinations of rational targeted agents.

**Conclusions:**

The early excitement about IGF-1R - targeted treatments has not translated into the clinical success that one might have anticipated. Certainly, undesirable side-effects arising from crosstalk with the InsR signaling pathway were a concern. Nevertheless, if the anti-tumor activity had been significant the issues with maintaining glucose homeostasis were likely manageable. The clinical studies testing various IGF-1R therapeutics did not stratify patients with any molecular markers. Further, by the time candidate predictive biomarkers were proposed and combinatorial treatment protocols designed to counter compensatory signaling pathways, pharmaceutical development of most IGF-1R targeting agents for oncology had ceased. Evidence summarized in this review support a re-examination of the use of IGF-1R sub-cellular localization as a biomarker in the selection of combination treatment regimens. Combining IGF-1R localization with other protein biomarkers, notably markers of replication stress (132), could enrich the patient population selected for IGF-1R targeted therapeutics. Additionally, this approach can help identify optimum combination drug regimens. A limitation of this approach is one shared by most immunohistochemistry (IHC) methods of biopsy analysis – IHC requires interpretation by expert pathologists and CLIA-certified protocols.

The intense interest in targeting IGF-1R has undoubtedly resulted in a large inventory of potential drugs that either never entered trials or were not pursued further when the disappointing data from unstratified trials emerged. A biomarker-informed re-evaluation of this inventory of drug candidates would be productive.

**List of abbreviations**

IGF-1R: Insulin-like growth factor receptor 1

IGF-2R: Insulin-like growth factor receptor 2

InsR: Insulin receptor

RTK: receptor tyrosine kinase

PI3K: phosphoinositide 3-kinase

AKT: also known as protein kinase B PKB

mTOR: mammalian target of rapamycin

RAS: a family of GTP-binding proteins, the name derives from “Rat sarcoma”

MAPK: mitogen-activated protein kinases

IGF-1, IGF-1: insulin-like growth factor 1 and 2

TSHR: thyrotropin receptor

GPCR: G-protein coupled receptor

PFS: progression-free survival

OS: overall survival

EFS: event-free survival

ATP: adenosine tri-phosphate

TKI: tyrosine kinase inhibitors

EGFR: epidermal growth factor receptor. Family includes other ErbB receptors

ERK: extracellular signal-related kinase

SFK: Src family kinase

CDK: cyclin-dependent kinase

IRS-1 and IRS-2: insulin receptor substrate 1 and 2

IGFBP: Insulin-like growth factor binding protein

mAb: monoclonal antibody

ICD: intracellular domain

FGFR1 and FGFR2: Fibroblast growth factor receptor 1 and 2

VEGFR1 and R2: vascular endothelial growth factor receptor-1 and -2

NLS: nuclear localization signal

DDT: DNA Damage Tolerance

RNAPol2: RNA polymerase 2

LEF-1: lymphoid enhancer binding factor1

TCF: T cell factor

ssDNA: single-stranded DNA

PCNA: proliferating cell nuclear antigen

RAD18: A ubiquitin ligase that ubiquitinates PCNA

DNA: deoxyribonucleic acid

GH: Growth hormone

NCT: National clinical trial

IHC: immunohistochemistry

CLIA: Clinical laboratory improvement amendments

**Declarations**

Ethics approval and consent to participate: Not applicable.

Consent for publication: Not applicable.

Accessibility of data and materials: Not applicable.

Competing interests: The authors declare that they have no competing interests.

Funding: This work was funded by National Institutes of Health grants RO1 CA207068 (to RSH) and R01 HL152094(RSH), Jeff Gordon’s Children’s Foundation (RSH)

Authors’ contributions: UKS did a literature analysis and wrote a summary that informed some sections. LJ inventoried pre-clinical studies of combination therapies to correlate with the clinical studies reported here. RSH wrote this review. All authors read and approved the final manuscript.

Acknowledgements: We thank Alex Krol for literature searches and assembling a table of IGF-1R targeted drugs and their in vitro activity. Kanksha Busch performed a literature survey on nuclear IGF-1R in Ewing sarcoma.

**Table 1:** IGF-1R targeted drugs evaluated in human trials.

| DRUG | CLASS |
| --- | --- |
|  |  |
| AMG479 (Ganitumab) | Human monoclonal antibody targeting IGF-1R |
| AVE1642 | Human monoclonal antibody targeting IGF-1R |
| BIIB022 | Human monoclonal antibody targeting IGF-1R |
| CP-751871 (figitumab) | Human monoclonal antibody targeting IGF-1R |
| IMCA12 (cixutumumab) | Human monoclonal antibody targeting IGF-1R |
| MK7454 (robatumumab) | Human monoclonal antibody targeting IGF-1R |
| MK0646 (dalotuzumab) | Human monoclonal antibody targeting IGF-1R |
| MM141 (istiratumab) | Human monoclonal bispecific antibody targeting IGF-1R and ErbB3 |
| RG1507 (teprotumumab) | Human monoclonal antibody targeting IGF-1R |
|  |  |
| AXL1717 (picropodophyllotoxin) | Small molecule non ATP-competitive IGF-1R kinase inhibitor |
| BMS-754807 | Small molecule ATP-competitive IGF-1R kinase inhibitor |
| KW-2450 | Small molecule IGF-1R and InsR kinase inhibitor |
| OSI906 (Linsitinib) | Small molecule ATP-competitive IGF-1R and InsR kinase inhibitor |
| PL225B | Small molecule IGF-1R kinase inhibitor |
| XL228 | Multi-targeted small molecule inhibitor of the IGF1R, Src, Abl tyrosine kinases |
|  |  |
| IGV-001 | Biologic-device; patient derived GBM cells treated with an antisense oligodeoxynucleotide towards IGF-1R |

**Table 2:** Common mechanisms of resistance to IGF-1R -targeted drugs

| **Mechanisms of resistance to IGF-1R targeted therapy** | **References** |
| --- | --- |
| Constitutive activation of downstream signaling PI3K-AKT-mTOR | (85, 140) |
| Constitutive activation of downstream signaling MAPK cascade | (141) |
| Absence of IRS-1, IRS-2 | (142) |
| Plasma IGF-1R sequestering of anti-IGF1R antibodies | (143) |
| InsR signaling compensating for IGF1R inhibition | (144) |
| InsR-IGF1R hybrid receptors | (28, 145) |
| Crosstalk with other receptors | (11, 74, 75, 146-149) |

**Table 3:** A sampling of the combinations of IGF-1R targeted drugs and other targeted agents that have entered clinical trials.

| **IGF-1R inhibitor** | **Co-targeted protein** | **Partner drug** | **Tumors** | **NCT Number** |
| --- | --- | --- | --- | --- |
|  |  |  |  |  |
| Ganitumab | mTOR | Evorolimus | Advanced cancer | NCT01061788 |
| R1507 | mTOR | Evorolimus | Advanced solid tumors | NCT00985374 |
| Cixutumumab | mTOR | Everolimus | Neuroendocrine carcinoma | NCT01204476 |
| Cixutumumab | mTOR | Everolimus | Solid tumors, NSCLC | NCT01061788 |
| Cixutumumab | mTOR | Temsirolumus | Pediatric solid tumors | NCT00880282 |
| Cixutumumab | mTOR | Temsirolumus | Sarcoma | NCT01614795 |
| Cixutumumab | mTOR | Temsirolumus | Sarcoma | NCT01016015 |
| Cixutumumab | mTOR | Temsirolumus | Metastatic prostate cancer | NCT01026623 |
| Cixutumumab | mTOR | Temsirolumus | Breast cancer | NCT00699491 |
|  |  |  |  |  |
| AVE1642 | proteasome | Bortezomib | Multiple myeloma | NCT01233895 |
|  |  |  |  |  |
| OSI-906 | EGFR | Erlotinib | Breast cancer | NCT01205685 |
| OSI-906 | EGFR | Erlotinib | Metastatic breast cancer | NCT01013506 |
| AVE1642 | EGFR | Erlotinib | Liver carcinoma | NCT00791544 |
| Cixutumumab | EGFR | Erlotinib | NSCLC | NCT00778167 |
| Cixutumumab | EGFR | Erlotinib | Pancreatic cancer | NCT00617708 |
| Ganitumab | HER-2 | Trastuzumab | Breast cancer | NCT01479179 |
| Cixutumumab | EGFR/ | Lapatinib | Breast cancer | NCT00684983 |
|  |  |  |  |  |
| Cixutumumab | MEK-1/2 | Selumetinib | Adult solid neoplasms | NCT01061749 |
|  |  |  |  |  |
| Ganitumab | SFK | Dasatinib | Rhabdomyosarcoma | NCT03041701 |
|  |  |  |  |  |
| Figitumumab | GH | Pegvisomant | Advanced solid tumors | NCT00976508 |
|  |  |  |  |  |
| Ganitumab | CDK4/6 | Pablociclib | Relapsed Ewsing sarcoma | NCT04129151 |
|  |  |  |  |  |
| BIIB022 | Kinases | Sorafenib | Hepatocellular carcinoma | NCT00956436 |
| AVE164 | Kinases | Sorafenib +Erlotinib) | Metastatic liver cancer | NCT00791544 |
|  |  |  |  |  |

**Figure Legends**

**Figure 1:** A schematic representation of IGF-1R protein architecture and the signaling cascades activated by the IGF-1R – IGF-1 interaction. Created with BioRender.com.

**Figure 2: IGF-1R and DNA Damage Tolerance**. Nuclear IGF-1R phosphorylates PCNA and promotes ubiquitination by RAD18. Ubiquitinated PCNA promotes recruitment of permissive polymerases to ssDNA breaks and stalled replication forks (markers of replication stress). Mono-ubiquitinated PCNA promotes translesion synthesis and error-prone lesion bypass. Poly-ubiquitinated PCNA promotes strand-switching and an error-free lesion bypass mechanism. In either case, the nuclear IGF-1R - PCNA pathway permits stalled replication to proceed allowing cells survival. Unrepaired ssDNA lesions would lead to double-stranded DNA breaks, and cell death. Hence nuclear IGF-1R promotes the survival of tumor cells under replication stress. Created with BioRender.com.

**REFERENCES**

1. Sawyers CL. Opportunities and challenges in the development of kinase inhibitor therapy for cancer. Genes Dev. 2003;17(24):2998-3010.

2. Baserga R. The contradictions of the insulin-like growth factor 1 receptor. Oncogene. 2000;19(49):5574-81.

3. Moxham CP, Duronio V, Jacobs S. Insulin-like growth factor I receptor beta-subunit heterogeneity. Evidence for hybrid tetramers composed of insulin-like growth factor I and insulin receptor heterodimers. The Journal of biological chemistry. 1989;264(22):13238-44.

4. Xu Y, Margetts MB, Venugopal H, Menting JG, Kirk NS, Croll TI, et al. How insulin-like growth factor I binds to a hybrid insulin receptor type 1 insulin-like growth factor receptor. Structure. 2022;30(8):1098-108 e6.

5. Mughal RS, Bridge K, Buza I, Slaaby R, Worm J, Klitgaard-Povlsen G, et al. Effects of obesity on insulin: insulin-like growth factor 1 hybrid receptor expression and Akt phosphorylation in conduit and resistance arteries. Diab Vasc Dis Res. 2019;16(2):160-70.

6. Bailyes EM, Nave BT, Soos MA, Orr SR, Hayward AC, Siddle K. Insulin receptor/IGF-I receptor hybrids are widely distributed in mammalian tissues: quantification of individual receptor species by selective immunoprecipitation and immunoblotting. The Biochemical journal. 1997;327 ( Pt 1)(Pt 1):209-15.

7. Baker J, Liu JP, Robertson EJ, Efstratiadis A. Role of insulin-like growth factors in embryonic and postnatal growth. Cell. 1993;75(1):73-82.

8. Belfiore A, Malaguarnera R, Vella V, Lawrence MC, Sciacca L, Frasca F, et al. Insulin Receptor Isoforms in Physiology and Disease: An Updated View. Endocr Rev. 2017;38(5):379-431.

9. Stanicka J, Rieger L, O'Shea S, Cox O, Coleman M, O'Flanagan C, et al. FES-related tyrosine kinase activates the insulin-like growth factor-1 receptor at sites of cell adhesion. Oncogene. 2018;37(23):3131-50.

10. Mauro L, Bartucci M, Morelli C, Ando S, Surmacz E. IGF-I receptor-induced cell-cell adhesion of MCF-7 breast cancer cells requires the expression of junction protein ZO-1. The Journal of biological chemistry. 2001;276(43):39892-7.

11. Riedemann J, Takiguchi M, Sohail M, Macaulay VM. The EGF receptor interacts with the type 1 IGF receptor and regulates its stability. Biochemical and biophysical research communications. 2007;355(3):707-14.

12. Krieger CC, Neumann S, Gershengorn MC. TSH/IGF1 receptor crosstalk: Mechanism and clinical implications. Pharmacol Ther. 2020;209:107502.

13. Rozengurt E, Sinnett-Smith J, Kisfalvi K. Crosstalk between insulin/insulin-like growth factor-1 receptors and G protein-coupled receptor signaling systems: a novel target for the antidiabetic drug metformin in pancreatic cancer. Clin Cancer Res. 2010;16(9):2505-11.

14. Resnicoff M, Abraham D, Yutanawiboonchai W, Rotman HL, Kajstura J, Rubin R, et al. The insulin-like growth factor I receptor protects tumor cells from apoptosis in vivo. Cancer research. 1995;55(11):2463-9.

15. Peruzzi F, Prisco M, Dews M, Salomoni P, Grassilli E, Romano G, et al. Multiple signaling pathways of the insulin-like growth factor 1 receptor in protection from apoptosis. Molecular and cellular biology. 1999;19(10):7203-15.

16. Cox OT, O'Shea S, Tresse E, Bustamante-Garrido M, Kiran-Deevi R, O'Connor R. IGF-1 Receptor and Adhesion Signaling: An Important Axis in Determining Cancer Cell Phenotype and Therapy Resistance. Front Endocrinol (Lausanne). 2015;6:106.

17. Kucab JE, Dunn SE. Role of IGF-1R in mediating breast cancer invasion and metastasis. Breast Dis. 2003;17:41-7.

18. Xie Y, Skytting B, Nilsson G, Brodin B, Larsson O. Expression of insulin-like growth factor-1 receptor in synovial sarcoma: association with an aggressive phenotype. Cancer research. 1999;59(15):3588-91.

19. Irie HY, Shrestha Y, Selfors LM, Frye F, Iida N, Wang Z, et al. PTK6 regulates IGF-1-induced anchorage-independent survival. PLoS One. 2010;5(7):e11729.

20. Bid HK, Zhan J, Phelps DA, Kurmasheva RT, Houghton PJ. Potent inhibition of angiogenesis by the IGF-1 receptor-targeting antibody SCH717454 is reversed by IGF-2. Mol Cancer Ther. 2012;11(3):649-59.

21. Li H, Batth IS, Qu X, Xu L, Song N, Wang R, et al. IGF-IR signaling in epithelial to mesenchymal transition and targeting IGF-IR therapy: overview and new insights. Mol Cancer. 2017;16(1):6.

22. Heskamp S, Boerman OC, Molkenboer-Kuenen JD, Wauters CA, Strobbe LJ, Mandigers CM, et al. Upregulation of IGF-1R expression during neoadjuvant therapy predicts poor outcome in breast cancer patients. PLoS One. 2015;10(2):e0117745.

23. Codony-Servat J, Cuatrecasas M, Asensio E, Montironi C, Martinez-Cardus A, Marin-Aguilera M, et al. Nuclear IGF-1R predicts chemotherapy and targeted therapy resistance in metastatic colorectal cancer. Br J Cancer. 2017;117(12):1777-86.

24. Li Y, Lu K, Zhao B, Zeng X, Xu S, Ma X, et al. Depletion of insulin-like growth factor 1 receptor increases radiosensitivity in colorectal cancer. J Gastrointest Oncol. 2020;11(6):1135-45.

25. Chitnis MM, Yuen JS, Protheroe AS, Pollak M, Macaulay VM. The type 1 insulin-like growth factor receptor pathway. Clin Cancer Res. 2008;14(20):6364-70.

26. Sciacca L, Mineo R, Pandini G, Murabito A, Vigneri R, Belfiore A. In IGF-I receptor-deficient leiomyosarcoma cells autocrine IGF-II induces cell invasion and protection from apoptosis via the insulin receptor isoform A. Oncogene. 2002;21(54):8240-50.

27. Vella V, Pandini G, Sciacca L, Mineo R, Vigneri R, Pezzino V, et al. A novel autocrine loop involving IGF-II and the insulin receptor isoform-A stimulates growth of thyroid cancer. J Clin Endocrinol Metab. 2002;87(1):245-54.

28. Belfiore A, Pandini G, Vella V, Squatrito S, Vigneri R. Insulin/IGF-I hybrid receptors play a major role in IGF-I signaling in thyroid cancer. Biochimie. 1999;81(4):403-7.

29. Avnet S, Sciacca L, Salerno M, Gancitano G, Cassarino MF, Longhi A, et al. Insulin receptor isoform A and insulin-like growth factor II as additional treatment targets in human osteosarcoma. Cancer research. 2009;69(6):2443-52.

30. Chettouh H, Fartoux L, Aoudjehane L, Wendum D, Claperon A, Chretien Y, et al. Mitogenic insulin receptor-A is overexpressed in human hepatocellular carcinoma due to EGFR-mediated dysregulation of RNA splicing factors. Cancer research. 2013;73(13):3974-86.

31. Vella V, Giuliano M, La Ferlita A, Pellegrino M, Gaudenzi G, Alaimo S, et al. Novel Mechanisms of Tumor Promotion by the Insulin Receptor Isoform A in Triple-Negative Breast Cancer Cells. Cells. 2021;10(11).

32. Belfiore A, Rapicavoli RV, Le Moli R, Lappano R, Morrione A, De Francesco EM, et al. IGF2: A Role in Metastasis and Tumor Evasion from Immune Surveillance? Biomedicines. 2023;11(1).

33. Vella V, Milluzzo A, Scalisi NM, Vigneri P, Sciacca L. Insulin Receptor Isoforms in Cancer. Int J Mol Sci. 2018;19(11).

34. Baserga R. The insulin-like growth factor I receptor: a key to tumor growth? Cancer research. 1995;55(2):249-52.

35. Aleksic T, Verrill C, Bryant RJ, Han C, Worrall AR, Brureau L, et al. IGF-1R associates with adverse outcomes after radical radiotherapy for prostate cancer. Br J Cancer. 2017;117(11):1600-6.

36. Dale OT, Aleksic T, Shah KA, Han C, Mehanna H, Rapozo DC, et al. IGF-1R expression is associated with HPV-negative status and adverse survival in head and neck squamous cell cancer. Carcinogenesis. 2015;36(6):648-55.

37. Alkhayyal N, Talaat I, Vinodnadat A, Maghazachi A, Abusnana S, Syrjanen K, et al. Correlation of Insulin-like Growth Factor 1 Receptor Expression With Different Molecular Subtypes of Breast Cancer in the UAE. Anticancer Res. 2020;40(3):1555-61.

38. Zheng Y, Wu C, Yang J, Zhao Y, Jia H, Xue M, et al. Insulin-like growth factor 1-induced enolase 2 deacetylation by HDAC3 promotes metastasis of pancreatic cancer. Signal Transduct Target Ther. 2020;5(1):53.

39. Sekharam M, Zhao H, Sun M, Fang Q, Zhang Q, Yuan Z, et al. Insulin-like growth factor 1 receptor enhances invasion and induces resistance to apoptosis of colon cancer cells through the Akt/Bcl-x(L) pathway. Cancer research. 2003;63(22):7708-16.

40. Xu J, Bie F, Wang Y, Chen X, Yan T, Du J. Prognostic value of IGF-1R in lung cancer: A PRISMA-compliant meta-analysis. Medicine (Baltimore). 2019;98(19):e15467.

41. Scotlandi K, Benini S, Sarti M, Serra M, Lollini PL, Maurici D, et al. Insulin-like growth factor I receptor-mediated circuit in Ewing's sarcoma/peripheral neuroectodermal tumor: a possible therapeutic target. Cancer research. 1996;56(20):4570-4.

42. Ameline B, Kovac M, Nathrath M, Barenboim M, Witt O, Krieg AH, et al. Overactivation of the IGF signalling pathway in osteosarcoma: a potential therapeutic target? J Pathol Clin Res. 2021;7(2):165-72.

43. Ma C, Wang Y, Wilson KM, Mucci LA, Stampfer MJ, Pollak M, et al. Circulating Insulin-Like Growth Factor 1-Related Biomarkers and Risk of Lethal Prostate Cancer. JNCI Cancer Spectr. 2022;6(1).

44. Lukanova A, Lundin E, Toniolo P, Micheli A, Akhmedkhanov A, Rinaldi S, et al. Circulating levels of insulin-like growth factor-I and risk of ovarian cancer. Int J Cancer. 2002;101(6):549-54.

45. Farabaugh SM, Boone DN, Lee AV. Role of IGF1R in Breast Cancer Subtypes, Stemness, and Lineage Differentiation. Front Endocrinol (Lausanne). 2015;6:59.

46. Shanmugalingam T, Bosco C, Ridley AJ, Van Hemelrijck M. Is there a role for IGF-1 in the development of second primary cancers? Cancer Med. 2016;5(11):3353-67.

47. Unger C, Kramer N, Unterleuthner D, Scherzer M, Burian A, Rudisch A, et al. Stromal-derived IGF2 promotes colon cancer progression via paracrine and autocrine mechanisms. Oncogene. 2017;36(38):5341-55.

48. Jentzsch V, Osipenko L, Scannell JW, Hickman JA. Costs and Causes of Oncology Drug Attrition With the Example of Insulin-Like Growth Factor-1 Receptor Inhibitors. JAMA Netw Open. 2023;6(7):e2324977.

49. Langer CJ, Novello S, Park K, Krzakowski M, Karp DD, Mok T, et al. Randomized, phase III trial of first-line figitumumab in combination with paclitaxel and carboplatin versus paclitaxel and carboplatin alone in patients with advanced non-small-cell lung cancer. J Clin Oncol. 2014;32(19):2059-66.

50. Sclafani F, Kim TY, Cunningham D, Kim TW, Tabernero J, Schmoll HJ, et al. A Randomized Phase II/III Study of Dalotuzumab in Combination With Cetuximab and Irinotecan in Chemorefractory, KRAS Wild-Type, Metastatic Colorectal Cancer. J Natl Cancer Inst. 2015;107(12):djv258.

51. Frappaz D, Federico SM, Pearson AD, Gore L, Macy ME, DuBois SG, et al. Phase 1 study of dalotuzumab monotherapy and ridaforolimus-dalotuzumab combination therapy in paediatric patients with advanced solid tumours. Eur J Cancer. 2016;62:9-17.

52. Fuchs CS, Azevedo S, Okusaka T, Van Laethem JL, Lipton LR, Riess H, et al. A phase 3 randomized, double-blind, placebo-controlled trial of ganitumab or placebo in combination with gemcitabine as first-line therapy for metastatic adenocarcinoma of the pancreas: the GAMMA trial. Ann Oncol. 2015;26(5):921-7.

53. DuBois SG, Krailo MD, Glade-Bender J, Buxton A, Laack N, Randall RL, et al. Randomized Phase III Trial of Ganitumab With Interval-Compressed Chemotherapy for Patients With Newly Diagnosed Metastatic Ewing Sarcoma: A Report From the Children's Oncology Group. J Clin Oncol. 2023;41(11):2098-107.

54. de Bono J, Lin CC, Chen LT, Corral J, Michalarea V, Rihawi K, et al. Two first-in-human studies of xentuzumab, a humanised insulin-like growth factor (IGF)-neutralising antibody, in patients with advanced solid tumours. Br J Cancer. 2020;122(9):1324-32.

55. Macaulay VM, Lord S, Hussain S, Maroto JP, Jones RH, Climent MA, et al. A Phase Ib/II study of IGF-neutralising antibody xentuzumab with enzalutamide in metastatic castration-resistant prostate cancer. Br J Cancer. 2023.

56. Schmid P, Cortes J, Joaquim A, Janez NM, Morales S, Diaz-Redondo T, et al. XENERA-1: a randomised double-blind Phase II trial of xentuzumab in combination with everolimus and exemestane versus everolimus and exemestane in patients with hormone receptor-positive/HER2-negative metastatic breast cancer and non-visceral disease. Breast Cancer Res. 2023;25(1):67.

57. von Mehren M, George S, Heinrich MC, Schuetze SM, Yap JT, Yu JQ, et al. Linsitinib (OSI-906) for the Treatment of Adult and Pediatric Wild-Type Gastrointestinal Stromal Tumors, a SARC Phase II Study. Clin Cancer Res. 2020;26(8):1837-45.

58. Fassnacht M, Berruti A, Baudin E, Demeure MJ, Gilbert J, Haak H, et al. Linsitinib (OSI-906) versus placebo for patients with locally advanced or metastatic adrenocortical carcinoma: a double-blind, randomised, phase 3 study. Lancet Oncol. 2015;16(4):426-35.

59. Ciuleanu TE, Ahmed S, Kim JH, Mezger J, Park K, Thomas M, et al. Randomised Phase 2 study of maintenance linsitinib (OSI-906) in combination with erlotinib compared with placebo plus erlotinib after platinum-based chemotherapy in patients with advanced non-small cell lung cancer. Br J Cancer. 2017;117(6):757-66.

60. Robertson JF, Ferrero JM, Bourgeois H, Kennecke H, de Boer RH, Jacot W, et al. Ganitumab with either exemestane or fulvestrant for postmenopausal women with advanced, hormone-receptor-positive breast cancer: a randomised, controlled, double-blind, phase 2 trial. Lancet Oncol. 2013;14(3):228-35.

61. Fayzullina D, Tsibulnikov S, Stempen M, Schroeder BA, Kumar N, Kharwar RK, et al. Novel Targeted Therapeutic Strategies for Ewing Sarcoma. Cancers (Basel). 2022;14(8).

62. Aiken R, Axelson M, Harmenberg J, Klockare M, Larsson O, Wassberg C. Phase I clinical trial of AXL1717 for treatment of relapsed malignant astrocytomas: analysis of dose and response. Oncotarget. 2017;8(46):81501-10.

63. Douglas RS, Kahaly GJ, Patel A, Sile S, Thompson EHZ, Perdok R, et al. Teprotumumab for the Treatment of Active Thyroid Eye Disease. N Engl J Med. 2020;382(4):341-52.

64. Gulbins A, Horstmann M, Daser A, Flogel U, Oeverhaus M, Bechrakis NE, et al. Linsitinib, an IGF-1R inhibitor, attenuates disease development and progression in a model of thyroid eye disease. Front Endocrinol (Lausanne). 2023;14:1211473.

65. Beckwith H, Yee D. Minireview: Were the IGF Signaling Inhibitors All Bad? Mol Endocrinol. 2015;29(11):1549-57.

66. Crudden C, Girnita A, Girnita L. Targeting the IGF-1R: The Tale of the Tortoise and the Hare. Front Endocrinol (Lausanne). 2015;6:64.

67. Wang P, Mak VC, Cheung LW. Drugging IGF-1R in cancer: New insights and emerging opportunities. Genes Dis. 2023;10(1):199-211.

68. Chen HX, Sharon E. IGF-1R as an anti-cancer target--trials and tribulations. Chin J Cancer. 2013;32(5):242-52.

69. King H, Aleksic T, Haluska P, Macaulay VM. Can we unlock the potential of IGF-1R inhibition in cancer therapy? Cancer Treat Rev. 2014;40(9):1096-105.

70. Buck E, Mulvihill M. Small molecule inhibitors of the IGF-1R/IR axis for the treatment of cancer. Expert Opin Investig Drugs. 2011;20(5):605-21.

71. Baserga R. The decline and fall of the IGF-I receptor. J Cell Physiol. 2013;228(4):675-9.

72. Lemmon MA, Schlessinger J. Cell signaling by receptor tyrosine kinases. Cell. 2010;141(7):1117-34.

73. Carlson JM, Doyle J. Highly optimized tolerance: robustness and design in complex systems. Phys Rev Lett. 2000;84(11):2529-32.

74. Kiely PA, O'Gorman D, Luong K, Ron D, O'Connor R. Insulin-like growth factor I controls a mutually exclusive association of RACK1 with protein phosphatase 2A and beta1 integrin to promote cell migration. Molecular and cellular biology. 2006;26(11):4041-51.

75. Ling Y, Maile LA, Clemmons DR. Tyrosine phosphorylation of the beta3-subunit of the alphaVbeta3 integrin is required for embrane association of the tyrosine phosphatase SHP-2 and its further recruitment to the insulin-like growth factor I receptor. Mol Endocrinol. 2003;17(9):1824-33.

76. Maile LA, Badley-Clarke J, Clemmons DR. The association between integrin-associated protein and SHPS-1 regulates insulin-like growth factor-I receptor signaling in vascular smooth muscle cells. Molecular biology of the cell. 2003;14(9):3519-28.

77. Hallak H, Seiler AE, Green JS, Ross BN, Rubin R. Association of heterotrimeric G(i) with the insulin-like growth factor-I receptor. Release of G(betagamma) subunits upon receptor activation. The Journal of biological chemistry. 2000;275(4):2255-8.

78. Buck E, Gokhale PC, Koujak S, Brown E, Eyzaguirre A, Tao N, et al. Compensatory insulin receptor (IR) activation on inhibition of insulin-like growth factor-1 receptor (IGF-1R): rationale for cotargeting IGF-1R and IR in cancer. Mol Cancer Ther. 2010;9(10):2652-64.

79. Xu L, Qi Y, Xu Y, Lian J, Wang X, Ning G, et al. Co-inhibition of EGFR and IGF1R synergistically impacts therapeutically on adrenocortical carcinoma. Oncotarget. 2016;7(24):36235-46.

80. Moran T, Felip E, Keedy V, Borghaei H, Shepherd FA, Insa A, et al. Activity of dalotuzumab, a selective anti-IGF1R antibody, in combination with erlotinib in unselected patients with Non-small-cell lung cancer: a phase I/II randomized trial. Exp Hematol Oncol. 2014;3(1):26.

81. Haluska P, Worden F, Olmos D, Yin D, Schteingart D, Batzel GN, et al. Safety, tolerability, and pharmacokinetics of the anti-IGF-1R monoclonal antibody figitumumab in patients with refractory adrenocortical carcinoma. Cancer Chemother Pharmacol. 2010;65(4):765-73.

82. Haluska P, Shaw HM, Batzel GN, Yin D, Molina JR, Molife LR, et al. Phase I dose escalation study of the anti insulin-like growth factor-I receptor monoclonal antibody CP-751,871 in patients with refractory solid tumors. Clin Cancer Res. 2007;13(19):5834-40.

83. Felice DL, El-Shennawy L, Zhao S, Lantvit DL, Shen Q, Unterman TG, et al. Growth hormone potentiates 17beta-estradiol-dependent breast cancer cell proliferation independently of IGF-I receptor signaling. Endocrinology. 2013;154(9):3219-27.

84. Arteaga CL, Osborne CK. Growth inhibition of human breast cancer cells in vitro with an antibody against the type I somatomedin receptor. Cancer research. 1989;49(22):6237-41.

85. Goncalves MD, Hopkins BD, Cantley LC. Phosphatidylinositol 3-Kinase, Growth Disorders, and Cancer. N Engl J Med. 2018;379(21):2052-62.

86. Fusco N, Sajjadi E, Venetis K, Gaudioso G, Lopez G, Corti C, et al. PTEN Alterations and Their Role in Cancer Management: Are We Making Headway on Precision Medicine? Genes (Basel). 2020;11(7).

87. Lee YR, Chen M, Pandolfi PP. The functions and regulation of the PTEN tumour suppressor: new modes and prospects. Nature reviews. 2018;19(9):547-62.

88. Tabernero J, Rojo F, Calvo E, Burris H, Judson I, Hazell K, et al. Dose- and schedule-dependent inhibition of the mammalian target of rapamycin pathway with everolimus: a phase I tumor pharmacodynamic study in patients with advanced solid tumors. J Clin Oncol. 2008;26(10):1603-10.

89. O'Reilly KE, Rojo F, She QB, Solit D, Mills GB, Smith D, et al. mTOR inhibition induces upstream receptor tyrosine kinase signaling and activates Akt. Cancer research. 2006;66(3):1500-8.

90. Wan X, Harkavy B, Shen N, Grohar P, Helman LJ. Rapamycin induces feedback activation of Akt signaling through an IGF-1R-dependent mechanism. Oncogene. 2007;26(13):1932-40.

91. Brana I, Berger R, Golan T, Haluska P, Edenfield J, Fiorica J, et al. A parallel-arm phase I trial of the humanised anti-IGF-1R antibody dalotuzumab in combination with the AKT inhibitor MK-2206, the mTOR inhibitor ridaforolimus, or the NOTCH inhibitor MK-0752, in patients with advanced solid tumours. Br J Cancer. 2014;111(10):1932-44.

92. Shulman DS, Merriam P, Choy E, Guenther LM, Cavanaugh KL, Kao PC, et al. Phase 2 trial of palbociclib and ganitumab in patients with relapsed Ewing sarcoma. Cancer Med. 2023;12(14):15207-16.

93. Akshintala S, Sundby RT, Bernstein D, Glod JW, Kaplan RN, Yohe ME, et al. Phase I trial of ganitumab plus dasatinib to cotarget the insulin-like growth factor 1 receptor and Src family kinase YES in rhabdomyosarcoma. Clin Cancer Res. 2023.

94. Cao H, Dong W, Shen H, Xu J, Zhu L, Liu Q, et al. Combinational Therapy Enhances the Effects of Anti-IGF-1R mAb Figitumumab to Target Small Cell Lung Cancer. PLoS One. 2015;10(8):e0135844.

95. Xue L, Chen F, Yue F, Camacho L, Kothapalli S, Wei G, et al. Metformin and an insulin/IGF-1 receptor inhibitor are synergistic in blocking growth of triple-negative breast cancer. Breast Cancer Res Treat. 2021;185(1):73-84.

96. La Thangue NB, Kerr DJ. Predictive biomarkers: a paradigm shift towards personalized cancer medicine. Nat Rev Clin Oncol. 2011;8(10):587-96.

97. Boone DN, Lee AV. Targeting the insulin-like growth factor receptor: developing biomarkers from gene expression profiling. Crit Rev Oncog. 2012;17(2):161-73.

98. Baserga R. The insulin receptor substrate-1: a biomarker for cancer? Exp Cell Res. 2009;315(5):727-32.

99. Schwartz GK, Tap WD, Qin LX, Livingston MB, Undevia SD, Chmielowski B, et al. Cixutumumab and temsirolimus for patients with bone and soft-tissue sarcoma: a multicentre, open-label, phase 2 trial. Lancet Oncol. 2013;14(4):371-82.

100. McCaffery I, Tudor Y, Deng H, Tang R, Suzuki S, Badola S, et al. Putative predictive biomarkers of survival in patients with metastatic pancreatic adenocarcinoma treated with gemcitabine and ganitumab, an IGF1R inhibitor. Clin Cancer Res. 2013;19(15):4282-9.

101. Yoneyama Y, Lanzerstorfer P, Niwa H, Umehara T, Shibano T, Yokoyama S, et al. IRS-1 acts as an endocytic regulator of IGF-I receptor to facilitate sustained IGF signaling. Elife. 2018;7.

102. Salani B, Passalacqua M, Maffioli S, Briatore L, Hamoudane M, Contini P, et al. IGF-IR internalizes with Caveolin-1 and PTRF/Cavin in HaCat cells. PLoS One. 2010;5(11):e14157.

103. Sehat B, Andersson S, Vasilcanu R, Girnita L, Larsson O. Role of ubiquitination in IGF-1 receptor signaling and degradation. PLoS One. 2007;2(4):e340.

104. Romanelli RJ, LeBeau AP, Fulmer CG, Lazzarino DA, Hochberg A, Wood TL. Insulin-like growth factor type-I receptor internalization and recycling mediate the sustained phosphorylation of Akt. The Journal of biological chemistry. 2007;282(31):22513-24.

105. Rieger L, O'Shea S, Godsmark G, Stanicka J, Kelly G, O'Connor R. IGF-1 receptor activity in the Golgi of migratory cancer cells depends on adhesion-dependent phosphorylation of Tyr(1250) and Tyr(1251). Sci Signal. 2020;13(633).

106. Aleksic T, Gray N, Wu X, Rieunier G, Osher E, Mills J, et al. Nuclear IGF1R Interacts with Regulatory Regions of Chromatin to Promote RNA Polymerase II Recruitment and Gene Expression Associated with Advanced Tumor Stage. Cancer research. 2018;78(13):3497-509.

107. Aleksic T, Chitnis MM, Perestenko OV, Gao S, Thomas PH, Turner GD, et al. Type 1 insulin-like growth factor receptor translocates to the nucleus of human tumor cells. Cancer research. 2010;70(16):6412-9.

108. Carpenter G, Liao HJ. Receptor tyrosine kinases in the nucleus. Cold Spring Harb Perspect Biol. 2013;5(10):a008979.

109. Sehat B, Tofigh A, Lin Y, Trocme E, Liljedahl U, Lagergren J, et al. SUMOylation mediates the nuclear translocation and signaling of the IGF-1 receptor. Sci Signal. 2010;3(108):ra10.

110. Lin SY, Makino K, Xia W, Matin A, Wen Y, Kwong KY, et al. Nuclear localization of EGF receptor and its potential new role as a transcription factor. Nat Cell Biol. 2001;3(9):802-8.

111. Wang SC, Lien HC, Xia W, Chen IF, Lo HW, Wang Z, et al. Binding at and transactivation of the COX-2 promoter by nuclear tyrosine kinase receptor ErbB-2. Cancer Cell. 2004;6(3):251-61.

112. Liu HS, Hsu PY, Lai MD, Chang HY, Ho CL, Cheng HL, et al. An unusual function of RON receptor tyrosine kinase as a transcriptional regulator in cooperation with EGFR in human cancer cells. Carcinogenesis. 2010;31(8):1456-64.

113. Stachowiak MK, Maher PA, Stachowiak EK. Integrative nuclear signaling in cell development--a role for FGF receptor-1. DNA Cell Biol. 2007;26(12):811-26.

114. Lee TH, Seng S, Sekine M, Hinton C, Fu Y, Avraham HK, et al. Vascular endothelial growth factor mediates intracrine survival in human breast carcinoma cells through internally expressed VEGFR1/FLT1. PLoS Med. 2007;4(6):e186.

115. Santos SC, Miguel C, Domingues I, Calado A, Zhu Z, Wu Y, et al. VEGF and VEGFR-2 (KDR) internalization is required for endothelial recovery during wound healing. Exp Cell Res. 2007;313(8):1561-74.

116. Packham S, Warsito D, Lin Y, Sadi S, Karlsson R, Sehat B, et al. Nuclear translocation of IGF-1R via p150(Glued) and an importin-beta/RanBP2-dependent pathway in cancer cells. Oncogene. 2015;34(17):2227-38.

117. Prisco M, Santini F, Baffa R, Liu M, Drakas R, Wu A, et al. Nuclear translocation of insulin receptor substrate-1 by the simian virus 40 T antigen and the activated type 1 insulin-like growth factor receptor. The Journal of biological chemistry. 2002;277(35):32078-85.

118. Warsito D, Lin Y, Gnirck AC, Sehat B, Larsson O. Nuclearly translocated insulin-like growth factor 1 receptor phosphorylates histone H3 at tyrosine 41 and induces SNAI2 expression via Brg1 chromatin remodeling protein. Oncotarget. 2016;7(27):42288-302.

119. Mills JV, Osher E, Rieunier G, Mills IG, Macaulay VM. IGF-1R nuclear import and recruitment to chromatin involves both alpha and beta subunits. Discov Oncol. 2021;12(1):13.

120. Bodzin AS, Wei Z, Hurtt R, Gu T, Doria C. Gefitinib resistance in HCC mahlavu cells: upregulation of CD133 expression, activation of IGF-1R signaling pathway, and enhancement of IGF-1R nuclear translocation. J Cell Physiol. 2012;227(7):2947-52.

121. Aslam MI, Hettmer S, Abraham J, Latocha D, Soundararajan A, Huang ET, et al. Dynamic and nuclear expression of PDGFRalpha and IGF-1R in alveolar Rhabdomyosarcoma. Mol Cancer Res. 2013;11(11):1303-13.

122. Lin Y, Liu H, Waraky A, Haglund F, Agarwal P, Jernberg-Wiklund H, et al. SUMO-modified insulin-like growth factor 1 receptor (IGF-1R) increases cell cycle progression and cell proliferation. J Cell Physiol. 2017;232(10):2722-30.

123. Warsito D, Sjostrom S, Andersson S, Larsson O, Sehat B. Nuclear IGF1R is a transcriptional co-activator of LEF1/TCF. EMBO Rep. 2012;13(3):244-50.

124. Waraky A, Lin Y, Warsito D, Haglund F, Aleem E, Larsson O. Nuclear insulin-like growth factor 1 receptor phosphorylates proliferating cell nuclear antigen and rescues stalled replication forks after DNA damage. The Journal of biological chemistry. 2017;292(44):18227-39.

125. Jamwal G, Singh G, Dar MS, Singh P, Bano N, Syed SH, et al. Identification of a unique loss-of-function mutation in IGF1R and a crosstalk between IGF1R and Wnt/beta-catenin signaling pathways. Biochim Biophys Acta Mol Cell Res. 2018;1865(6):920-31.

126. Sarfstein R, Pasmanik-Chor M, Yeheskel A, Edry L, Shomron N, Warman N, et al. Insulin-like growth factor-I receptor (IGF-IR) translocates to nucleus and autoregulates IGF-IR gene expression in breast cancer cells. The Journal of biological chemistry. 2012;287(4):2766-76.

127. Baatar S, Bai T, Yokobori T, Gombodorj N, Nakazawa N, Ubukata Y, et al. High RAD18 Expression is Associated with Disease Progression and Poor Prognosis in Patients with Gastric Cancer. Annals of surgical oncology. 2020;27(11):4360-8.

128. Wu B, Wang H, Zhang L, Sun C, Li H, Jiang C, et al. High expression of RAD18 in glioma induces radiotherapy resistance via down-regulating P53 expression. Biomed Pharmacother. 2019;112:108555.

129. Zhu X, Zou S, Zhou J, Zhu H, Zhang S, Shang Z, et al. REV3L, the catalytic subunit of DNA polymerase zeta, is involved in the progression and chemoresistance of esophageal squamous cell carcinoma. Oncol Rep. 2016;35(3):1664-70.

130. Zou S, Shang ZF, Liu B, Zhang S, Wu J, Huang M, et al. DNA polymerase iota (Pol iota) promotes invasion and metastasis of esophageal squamous cell carcinoma. Oncotarget. 2016;7(22):32274-85.

131. Kotsantis P, Petermann E, Boulton SJ. Mechanisms of Oncogene-Induced Replication Stress: Jigsaw Falling into Place. Cancer Discov. 2018;8(5):537-55.

132. Soni UK, Wang Y, Pandey RN, Roberts R, Pressey JG, Hegde RS. Molecularly Defined Subsets of Ewing Sarcoma Tumors Differ in Their Responses to IGF1R and WEE1 Inhibition. Clin Cancer Res. 2023;29(2):458-71.

133. Molina ER, Chim LK, Lamhamedi-Cherradi SE, Mohiuddin S, McCall D, Cuglievan B, et al. Correlation of nuclear pIGF-1R/IGF-1R and YAP/TAZ in a tissue microarray with outcomes in osteosarcoma patients. Oncotarget. 2022;13:521-33.

134. Clement F, Martin A, Venara M, de Lujan Calcagno M, Matho C, Maglio S, et al. Type 1 IGF Receptor Localization in Paediatric Gliomas: Significant Association with WHO Grading and Clinical Outcome. Horm Cancer. 2018;9(3):205-14.

135. Palmerini E, Benassi MS, Quattrini I, Pazzaglia L, Donati D, Benini S, et al. Prognostic and predictive role of CXCR4, IGF-1R and Ezrin expression in localized synovial sarcoma: is chemotaxis important to tumor response? Orphanet J Rare Dis. 2015;10:6.

136. van Gaal JC, Roeffen MH, Flucke UE, van der Laak JA, van der Heijden G, de Bont ES, et al. Simultaneous targeting of insulin-like growth factor-1 receptor and anaplastic lymphoma kinase in embryonal and alveolar rhabdomyosarcoma: a rational choice. Eur J Cancer. 2013;49(16):3462-70.

137. Asmane I, Watkin E, Alberti L, Duc A, Marec-Berard P, Ray-Coquard I, et al. Insulin-like growth factor type 1 receptor (IGF-1R) exclusive nuclear staining: a predictive biomarker for IGF-1R monoclonal antibody (Ab) therapy in sarcomas. Eur J Cancer. 2012;48(16):3027-35.

138. Cybulla E, Vindigni A. Leveraging the replication stress response to optimize cancer therapy. Nat Rev Cancer. 2023;23(1):6-24.

139. Konstantinopoulos PA, da Costa A, Gulhan D, Lee EK, Cheng SC, Hendrickson AEW, et al. A Replication stress biomarker is associated with response to gemcitabine versus combined gemcitabine and ATR inhibitor therapy in ovarian cancer. Nat Commun. 2021;12(1):5574.

140. Patel M, Gomez NC, McFadden AW, Moats-Staats BM, Wu S, Rojas A, et al. PTEN deficiency mediates a reciprocal response to IGFI and mTOR inhibition. Mol Cancer Res. 2014;12(11):1610-20.

141. Buck E, Eyzaguirre A, Rosenfeld-Franklin M, Thomson S, Mulvihill M, Barr S, et al. Feedback mechanisms promote cooperativity for small molecule inhibitors of epidermal and insulin-like growth factor receptors. Cancer research. 2008;68(20):8322-32.

142. Byron SA, Horwitz KB, Richer JK, Lange CA, Zhang X, Yee D. Insulin receptor substrates mediate distinct biological responses to insulin-like growth factor receptor activation in breast cancer cells. Br J Cancer. 2006;95(9):1220-8.

143. Xu JW, Wang TX, You L, Zheng LF, Shu H, Zhang TP, et al. Insulin-like growth factor 1 receptor (IGF-1R) as a target of MiR-497 and plasma IGF-1R levels associated with TNM stage of pancreatic cancer. PLoS One. 2014;9(3):e92847.

144. Weinstein D, Sarfstein R, Laron Z, Werner H. Insulin receptor compensates for IGF1R inhibition and directly induces mitogenic activity in prostate cancer cells. Endocr Connect. 2014;3(1):24-35.

145. Pandini G, Vigneri R, Costantino A, Frasca F, Ippolito A, Fujita-Yamaguchi Y, et al. Insulin and insulin-like growth factor-I (IGF-I) receptor overexpression in breast cancers leads to insulin/IGF-I hybrid receptor overexpression: evidence for a second mechanism of IGF-I signaling. Clin Cancer Res. 1999;5(7):1935-44.

146. Crudden C, Ilic M, Suleymanova N, Worrall C, Girnita A, Girnita L. The dichotomy of the Insulin-like growth factor 1 receptor: RTK and GPCR: friend or foe for cancer treatment? Growth Horm IGF Res. 2015;25(1):2-12.

147. Kiely PA, Sant A, O'Connor R. RACK1 is an insulin-like growth factor 1 (IGF-1) receptor-interacting protein that can regulate IGF-1-mediated Akt activation and protection from cell death. The Journal of biological chemistry. 2002;277(25):22581-9.

148. Manes S, Mira E, Gomez-Mouton C, Zhao ZJ, Lacalle RA, Martinez AC. Concerted activity of tyrosine phosphatase SHP-2 and focal adhesion kinase in regulation of cell motility. Molecular and cellular biology. 1999;19(4):3125-35.

149. Delcourt N, Bockaert J, Marin P. GPCR-jacking: from a new route in RTK signalling to a new concept in GPCR activation. Trends Pharmacol Sci. 2007;28(12):602-7.
