# Supplementary material for: IGF-1R targeting in cancer – does sub-cellular localization matter?
Source: J Exp Clin Cancer Res. 2023 Oct 20;42:273. doi: 10.1186/s13046-023-02850-7 (PMC10588251; doi:10.1186/s13046-023-02850-7)
Supplement: Supplementary file 1 — Supplementary Material 1 [file 13046_2023_2850_MOESM1_ESM.docx]

We thank the reviewers for a very thorough examination of the manuscript. Their comments have greatly improved the document and we trust that we have incorporated all their suggestions.

Specific responses to comments are below each comment.

**REVIEWER 1**

1) Introduction: it is limited and lack important data. It would be important to discuss some historic

published data from Baserga's laboratory, which really established the IGF-IR as possible target in cancer.

*We have added a section specifically called IGF-1R in cancer (pages 4 – 5) and included extensive reference to work from the Baserga lab.*

2) The authors did not mention the two isoforms of the Insulin receptor, IR-A and IR-B and the fact that

IGF2 activates not only the IGF-IR but also the IR-A, which can also dimerizes with the IGF-IR, and these

hybryds are activated by all IGFs ligands.

&

3) Another point missing is the fact that the IR-A is strongly mitogenic as compared to the IR-B, which

mostly regulates metabolisms, as reported in the review.

*Page 4, line 6 – mention of IGF-2 binding IR-A*

*Two forms of IR, their dimerization properties and the fact that IR-A is mitogenic is mentioned on page 5, lines 4 – 8.*

4) Many sections are not supported by any reference cited.

*22 more references have been added where appropriate and reasonable – for a total of 149 references.*

5) "indeed, the ATP-binding domains are identical" Homology is high, but the IGF-IR and IR kinase domains are not exactly identical.

*This sentence has been modified, page 6 line 16*

6) There are evidences of compensatory role of IR-A when IGF-1R is disrupted. These data are not

discussed.

*Mentioned with reference on page 7, line 15.*

7) As stated by the authors, the major theme of the review is IGF-IR subcellular localization and its

relevance for the clinic as biomarker of response. This concept has to be expanded.

*Pages 9 – 13 focus on this topic.*

**REVIEWER 2**

Major points:

1.Pag. 4 lines 11-18: it is stated that IGF2 can bind IGF2R, IGF1R and the hybrid InsR/IGF1R. The Authors

should mention that IGF2 is a bona fide ligand for the isoform A of the insulin receptor, as well. Considering the important cross-talk between IGF1R and IR and the important role of the IR isoform A in cancer, the Authors should add this information to strengthen their review (doi: 10.3390/cells10113145; doi:10.3390/biomedicines11010229).

*IGF-2 binding to IR-A is mentioned on page 4 line 6.*

*The references listed in this comment to support a role for IR-A in cancer have been included on page 5, line 6.*

2. Pag. 4 lines 18-29: statements in here are only partially correct, as IGF1R and IR overlap also in mediating proliferative actions and in associating with transduction partners. In fact, while it's true that (i) IGF1R mediates proliferative effects and (ii) IGF1R binds directly to certain transduction partners, it is also true that

(i) the isoform A of the insulin receptor exhibits strong mitogenic effects (doi: 10.3390/cells10113145), and

(II) InsR has also been shown to associate directly to certain molecular partners (doi: 10.1186/s13058-023-01686-5; DOI: 10.3390/biom11070926). The Authors should add this missing information and reformulate their sentences.

*The mitogenic effects of IR-A and it’s association with cancer have been referred to on page 5, lines 4 – 8. The requested references to work from the Belfiore group are now included.*

3. Pag. 4-5: "IGF-1R signaling promotes numerous cancer-associated phenotypes such as growth, metastasis, and anchorage-independent growth. Accordingly, there is a strong positive association between high levels of IGF-1R pathway signaling and cancer (though activating mutations of the IGF1R gene have not been reported). These two sentences need appropriate references. Furthermore, it is important to state the role of IGF1R in cancer cell metabolism, angiogenesis and resistance to therapy, as these are very well known (10.1038/bjc.2017.279; doi: 10.3390/cancers13040621; doi: 10.1210/endrev/bnad005; doi: 10.1186/s13058-017-0923-5).

*Appropriate references have been included in the re-formulated sections including a reference to a potential role in tumor angiogenesis (page 4 lines 23-25, page 5 lines 1-4)*

4. To facilitate the readers, the Authors should include a table of agents targeting IGF1R tested in clinical trials for diverse types of cancer and the respective outcomes.

*Table 1 lists IGF-1R-targeting agents used in human trials. The outcomes of these trials are described on pages 5 – 6.*

5. In the conclusion section, the Authors should focus on what is missing and which could be the potential obstacles and novel strategies to increase the feasibility of anti-IGF1R strategies in the clinic.

*On page 14 (lines 11 – 18) we have included more detail on obstacles to the use of IGF-1R localization as a biomarker – the focus of this review. We have also included references of several more general reviews of anti-IGF1R strategies throughout the manuscript.*

Minor points:

1. The word insulin is repeated two times in line 11, pag. 4.

*Thank-you! This has been fixed.*

2. Line 28 pag 8 rephrase: menormin (which also COULD has have anticancer activity). Certain recent clinical trials have demonstrated ineffectiveness of menormin (doi: 10.1001/jama.2022.6147).

*This phrase has been removed – page 8.*
